# Family Members Additively Repress the Ectopic Expression of BASIC PENTACYSTEINE3 to Prevent Disorders in Arabidopsis Circadian Vegetative Development

**DOI:** 10.3389/fpls.2022.919946

**Published:** 2022-05-26

**Authors:** Yi-Chen Lee, Pei-Ting Tsai, Xun-Xian Huang, Huang-Lung Tsai

**Affiliations:** Institute of Molecular and Cellular Biology, National Taiwan University, Taipei, Taiwan

**Keywords:** *Arabidopsis thaliana*, BPC transcription factor, TCP transcription factor, circadian clock, leaf development

## Abstract

BARLEY B-RECOMBINANT/BASIC PENTACYSTEINE (BBR/BPC) family members are plant-specific GAGA-motif binding factors (GAFs) controlling multiple developmental processes of growth and propagation. BPCs recruit histone remodeling factors for transcriptional repression of downstream targets. It has been revealed that BPCs have an overlapping and antagonistic relationship in regulating development. In this study, we showed disturbances interfering with the homeostasis of *BPC* expressions impede growth and development. The ectopic expression of *BPC3* results in the daily growth defect shown by higher-order *bpc* mutants. Oscillations of multiple circadian clock genes are phase-delayed in the quadruple mutant of *bpc1 bpc2 bpc4 bpc6* (*bpc1,2,4,6*). By introducing the overexpression of BPC3 into wild-type Arabidopsis, we found that BPC3 is a repressor participating in its repression and repressing multiple regulators essential to the circadian clock. However, the induction of BPC3 overexpression did not fully replicate clock defects shown by the quadruple mutant, indicating that in addition to the *BPC3* antagonization, BPC members also cofunction in the circadian clock regulation. A leaf edge defect similar to that shown by *bpc1,2,4,6* is also observed under *BPC3* induction, accompanied by repression of a subset of *TCPs* required for the edge formation. This proves that *BPC3* is a repressor that must be confined during the vegetative phase. Our findings demonstrate that BPCs form a meticulous repressor network for restricting their repressive functions to molecular mechanisms controlling plant growth and development.

## Introduction

Plant-specific GAGA-binding factors, BASIC PENTACYSTEINEs (BPCs; also called BARLEY B RECOMBINANTs, BBRs), are transcription factors binding to the GA-dinucleotide repeats (Sangwan and O’Brian, 2002; Santi et al., 2003; Meister et al., 2004; Kooiker et al., 2005; Simonini et al., 2012; Hecker et al., 2015) frequently found in promoters of Arabidopsis genes (Monfared et al., 2011; Hecker et al., 2015). Coincident with the prevalence of GA-repeats in the Arabidopsis genome, BPCs regulate wide-ranged homeotic genes for the maintenance of the shoot apical meristem size (Simonini and Kater, 2014), root development (Monfared et al., 2011; Mu et al., 2017), and developmental transition of the ovule (Meister et al., 2004; Kooiker et al., 2005; Monfared et al., 2011; Simonini et al., 2012; Wu et al., 2020). In Arabidopsis, BPC members are categorized into three subclasses based on protein similarity: BPC1, BPC2, and BPC3 in class I; BPC4, the pseudogene BPC5, and BPC6 in class II; and BPC7 in class III (Meister et al., 2004; Monfared et al., 2011). Each class of BPC functions *via* targeting class-specific downstream genes. For example, class I members, BPC1 and BPC2, recruit polycomb repressive complex 2 (PRC2) to negatively regulate the spatiotemporal transcription of *FUSCA3* in reproductive organs (Wu et al., 2020). Multiple homeotic genes targeted by class I BPC are not significantly changed in mutants related to class II BPC, *bpc4 bpc6* or *lhp1-4 bpc4 bpc6* (Hecker et al., 2015), supporting that BPCs conduct class-specific functions (Hecker et al., 2015). However, the high-order mutants showing pleiotropic phenotypes, such as *bpc1-1 bpc2* (*bpc1,2*) and *bpc1-1 bpc2 bpc4 bpc6* (*bpc1,2,4,6*), can be partially rescued by the mutation of *BPC3*. This indicates that classes I and II BPCs (BPC1, BPC2, BPC4, and BPC6) also have overlapping roles in antagonizing *BPC3* (Monfared et al., 2011). In addition, it was found that BPCs of classes I and II act redundantly in a general regulatory complex composed of BPCs, MADS-domain factors, and PRCs to confine the homeotic gene *SEEDSTICK* (*STK*) expression (Petrella et al., 2020), indicating that the redundancy is not restricted among BPC members belonging to the same subclass.

Developmental defects observed in the *BPC* mutants, including the defective elongations of the hypocotyl (Dowson-Day and Millar, 1999), petiole (Engelmann and Johnsson, 1998) and inflorescence stem (Jouve et al., 1998), and flowering time control (Shim et al., 2017), are phenotypes commonly shown by circadian clock mutants. The circadian clock in Arabidopsis is composed of the double-negative feedback loop formed by *CIRCADIAN CLOCK ASSOCIATED1* (*CCA1*)/*LATE ELONGATED HYPOCOTYL* (*LHY*) and *TIMING OF CAB EXPRESSION1* (*TOC1*; Harmer et al., 2000; Alabadi et al., 2001). The peak expression of the morning genes *CCA1/LHY* at dawn represses the evening gene *TOC1* (Alabadi et al., 2001), whereas *TOC1* reciprocally represses *CCA1/LHY* at dusk (Pokhilko et al., 2010, 2012; Gendron et al., 2012; Huang et al., 2012). Besides TOC1, the expression of the *CCA1* during the day can be consecutively repressed by negative regulators PSEUDORESPONSE REGULATOR9 (PRR9), PRR7, PRR5, and CCA1 HIKING EXPEDITION (CHE) from the morning to midnight (Nakamichi et al., 2005, 2010; Pruneda-Paz et al., 2009). Most circadian clock genes encode transcription factors functioning in gene repression. A previous study has shown that the induction of TOC1 upregulates a subset of genes encompassing sequence patterns of GAGA motifs at the promoter regions (Gendron et al., 2012). This indicates that BPCs might regulate the circadian phenotypes with the clock components.

Significant defects showing curled adult leaves are seen in *BPC* mutants’ rosettes (Monfared et al., 2011), revealing that BPCs play regulatory roles for leaf development. A subset of *TEOSINTE BRANCHED1*, *CYCLOIDEA*, *PROLIFERATING CELL FACTOR* (*TCP*) transcription factors is involved in leaf development *via* the overlap of suppressing the serrated edge of adult leaves (Koyama et al., 2010). The exaggerated or deficient activity of the *TCP* subset results in disorders of leaf edge formation (Koyama et al., 2017). In addition to the transcriptional regulation, the *TCP* genes are targeted by miR319, which restrains *TCP* mRNA abundance in an acceptable range at the posttranscriptional stage (Palatnik et al., 2003; Koyama et al., 2017; Jiang et al., 2018). It has been shown that the binding motifs of TCP transcription factors overlap with the DNA-binding properties of BPC6 in addition to GAGA motifs (Shanks et al., 2018), implying that TCPs and BPCs may coordinate the downstream genes in leaf morphology controls.

In this study, we expose the function of *BPC3* is transcriptionally repressed by overlapping BPC members during the vegetative phase. BPC3 is involved in regulating its homeostasis of transcript level with other BPCs. *BPC3* repression is disrupted by *BPC* mutations or *via* introducing BPC3 overexpression broadly interferes with mechanisms, including circadian clock and leaf morphology during vegetative growth. These results suggest that BPC3 is a part of the complex BPC-repressive network confining downstream genes from the exaggerated expression during the vegetative stage.

## Materials and Methods

### Plant Materials and Growth Conditions

Plants used in this study were under *Arabidopsis thaliana* Columbia-0 (Col-0) background. Seeds of *bpc4* and *bpc1-1 bpc2 bpc4 bpc6* were gifts from Charles Gasser (Monfared et al., 2011). Mutant seeds of *bpc1-1 bpc2* (CS68700) and *bpc1-1 bpc2 bpc3-1* (CS68699) were obtained from Arabidopsis Biological Resource Center (ABRC), and genotypes were validated as previously described (Monfared et al., 2011). Wild-type and mutant seeds were germinated on half-strength Murashige and Skoog medium (Murashige and Skoog, 1962) solidified by 0.8% phytoagar and stratified at 4°C for 3 days under dark. Seedlings grown by white light illumination (75–100 μmol m^–2^ s^–1^) at 22°C under a photoperiod of 16-h light/8-h dark or 12-h light/12-h dark for later assessing the gene profiles under continuous light of free-running conditions were used for RNA preparations.

### Rosette Area Expansion Analysis

Plant seedlings were grown in soil pots at 22°C under 16 h light/8 h dark at a fluence rate of 45–55 μmol m^–2^ s^–1^. The plant’s growth was monitored in a temperature- and light-controlled chamber (Taiwan Hipoint). Lights were produced by LED lamps of 470 ± 30, 560 ± 20, and 660 ± 25 nm output at a 28:14:100 ratio. The fluence rate was measured by using the LI-250 radiometer (LI-COR). Color images of plant growth were taken hourly and processed by preserving green color to represent the rosette area. The rosette area was outlined and measured using the “Wand (tracing) tool” of ImageJ 1.53c on the processed image. The data of the rosette area were averaged across the growth process using a 10-ZT sliding window, and the area expanded per hour was calculated.

### DNA Constructions

The fragments of XVE, BAR, the gene of interest, and the EYFP-HA or HA tag generated by PCR were purified and assembled to the pER8 binary vector backbone. The *BPC1*, *BPC3*, and *BPC4* cDNAs were amplified using Phusion High-Fidelity DNA polymerase (NEB) from cDNAs generated from Arabidopsis rosette leaves and subcloned for use as templates in different destination PCRs. The synthetic XVE gene driven by G10-90 was amplified from pER8. The Pro_MAS_-BAR-Ter_MAS_ was amplified from the binary vector pEarleyGate 100. The *EYFP* gene was amplified from a template derived from the pEYFP plasmid. HA tag possessing triple tandem HA (3 × HA) was fill-in generated using 27-nt-paired DNA oligos. The amplified *BPC3* and *3* × *HA* fragments were assembled with an *Asc*I-*Spe*I linearized pER8 backbone fragment by using NEBuilder HiFi DNA Assembly Master Mix (NEB) to generate the construct *XVE:BPC3-HA*. The assembled plasmid was linearized by *Hin*dIII and *Asc*I and used as the backbone to assemble with amplified *G10-90*, *XVE*, *BAR* fragments and further modified as the Bar-resistant binary vector, *XVE:BPC3-HA*. The *XVE:BPC4-HA*, *XVE:BPC3-EYFP-HA*, and *XVE:BPC4-EYFP-HA* constructs were generated using the *Xho*I-*Spe*I linearized XVE:BPC3-HA as the backbone for assembling with *BPC3* or *BPC4*, *EYFP*, and *3* × *HA* fragments. Forward and reverse primers of interest for DNA amplification are listed in Supplementary Table 1.

### RNA Preparation and Quantitative Real-Time PCR

As described below, four to six seedlings grown under the indicated entrainment or free-running conditions were harvested at indicated age and time for total RNA isolation by using the pine tree method (Chang et al., 1993). Seedlings were ground into powder in liquid nitrogen and extracted by 700 μl pine-tree buffer (2% CTAB, 2% PVP, 2M NaCl, 0.5g/L spermidine, 25 mM EDTA, 100 mM Tris pH 8.0, and 2% 2-mercaptoethanol) at 65°C for 5 min. The RNA mixture was extracted with 450 μl of chloroform-isoamyl alcohol (24:1). The aqueous phase of the extraction was saved in 2M LiCl to precipitate RNA at 4°C for overnight. According to the manufacturer’s instruction, two micrograms of prified RNA were used to synthesize cDNA by conducting reverse transcription with Superscript II reverse transcriptase package (Invitrogen). The quantitative real-time PCR reaction was prepared using qPCRBIO SyGreen Mix (Cat. No. PB20.11, PCRBIOSYSTEMS) for performing qPCR in MA-6000 Real-Time Quantitative Thermal Cycler (Molarray). The gene expression relative to the internal control *UBQ10* was determined by using the comparative threshold cycle (C_T_) method. Primers used for the qPCR assays are listed in Supplementary Table 1.

### Immunoblot Assays

Total protein was extracted from transiently infected seedlings of AGROBEST or transgenic lines under the indicated conditions in 1 × Laemmli sample buffer (60 mM Tris–HCl pH 6.8, 10% glycerol, 1% SDS, 1% β-mercaptoethanol, and 0.01% bromophenol blue). Protein lysates were separated by 10% SDS-polyacrylamide gel electrophoresis in the Tris-glycine running system and transferred to a polyvinylidene fluoride (PVDF) membrane (Perkin Elmer) for signal detection. HA-tagged proteins were detected using the mouse monoclonal anti-HA antibody (H3663; Sigma-Aldrich).

### Chromatin Immunoprecipitation qPCR Assays

Rosette leaves of 22-day-old transgenic plants of *XVE:BPC3-EYFP-HA* lines grown under 16-h light/8-h dark cycles were cut at ZT9 and floated on half-strength MS solution containing 0 or 50 μM 17-β-estradiol to induce BPC3-EYFP-HA expression for 24 h. The leaf samples were used in Chromatin immunoprecipitation (ChIP) assays as described previously (Wang et al., 2011) with modifications. In brief, the leaves were crosslinked with fixation buffer (0.4 M sucrose, 10 mM Tris-HCl pH 8.0, 1 mM PMSF, 1 mM EDTA, 1% formaldehyde) under vacuum for 20 min and stopped in 125 mM glycine. Leaf materials were ground into powder in liquid nitrogen and lysed 500 μl powder by 800 μl nuclei lysis buffer (50 mM HEPES pH 7.5, 0.1% sodium deoxycholate, 0.5% SDS, 150 mM NaCl, 1% Triton X-100, 0.1 mM PMSF, 1 × Roche protease inhibitor cocktail). The lysate was filtered using a 100-μm nylon mesh. Seven hundred μl of the filtrate with two 3-mm glass beads was sonicated by 70 cycles of 20-sec-on/20-sec-off in a 2-ml tube with the instrument S2 focused-ultrasonicator (Covaris) setting parameters as duty cycle of 20%, intensity of 4, cycles per burst 4, and bath temperature of 7°C to shear chromatins in a length of approximately 0.5 kb. A one-tenth volume of sonicated lysate was saved as the input fraction. Chromatin complexes in the lysate were caught overnight at 4°C by anti-HA magnetic beads (monoclonal clone CB051, Origene) pre-equilibrated by 1 μg/ml salmon sperm DNA and 1 mg/ml BSA. The magnetic beads were washed three times by nuclei lysis buffer, three times by LNDET buffer (0.25 LiCl, 1% Nonidet P-40, 1% sodium deoxycholate, 1 mM EDTA), and three times by TE buffer (10 mM Tris-HCl pH 8.0, 1 mM EDTA). Chromatin complexes immunoprecipitated on beads were eluted by the elution buffer (0.5% SDS, 0.1 M NaHCO_3_). To release DNA, the cross-linked chromatin complexes were eluted, and input fractions were digested with 0.25 μg/μl Proteinase-K (Boehringer Mannheim) at 65°C overnight. The DNA was purified by using the QIAEXII gel purification kit (Qiagen), and the amount was determined by qPCR with corresponding primers. Primers used in ChIP-qPCR are listed in Supplementary Table 1.

## Results

### Growth Defects of *bpc* Mutants Are Anticorrelated With *BPC3* Transcript Level

**FIGURE 1:**
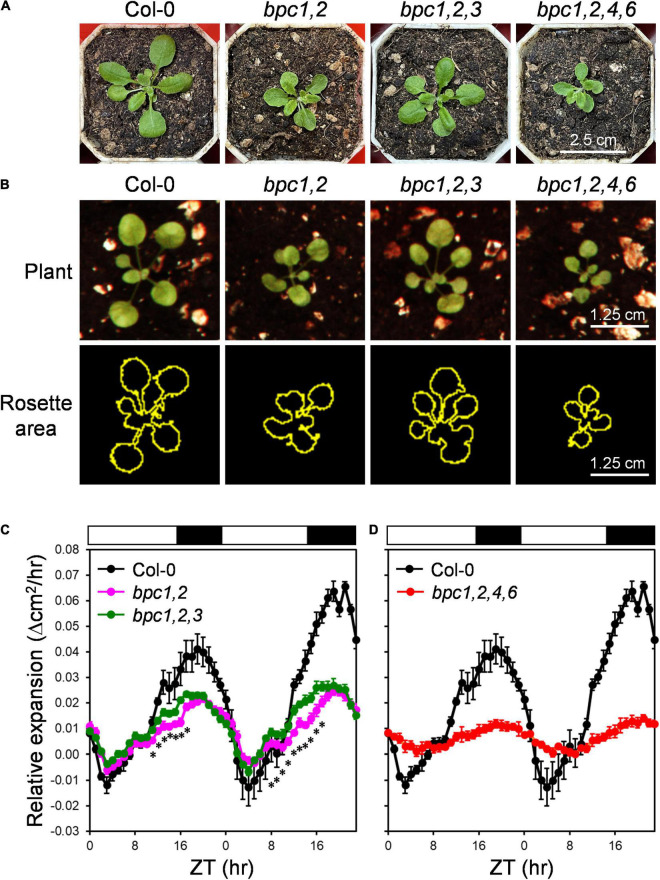
*BPC3* antagonized the circadian growth during the vegetative stage. **(A)** Plants representing wild-type (Col-0), *bpc1,2*, *bpc1,2,3* and *bpc1,2,4,6* grown under the long-day condition (16-h light/8-h dark, 45–55 mmol m^–2^ s^–1^) was photographed on day 21 for the size comparison. **(B)** The hour-growth of the indicated plants was image recorded (upper panel) and outlined by using the tracing tool of ImageJ 1.53c (yellow outlines, lower panel) to project the rosette area. **(C,D)** Relative expansion of rosette area was measured for each plant line from zeitgeber time 0 (ZT 0) on day 14 to ZT 23 on day 15 based on pixel quantitation of plant outlines. Rosette area across ZT points with a 10-ZT sliding window was averaged to lower leaf movement and nutation effects on the area. Data are mean ± S.E. (*n* = 7–9). Asterisks indicate ZTs on which the expansion rate of *bpc1,2,3* was significantly different from that of *bpc1,2* (Student’s *t-*test; *P < 0.01; *n* = 9). The expansion rate of Col-0 was duplicated in **(C,D)** for comparisons with *bpc* mutantsin plot charts. White and black bars indicate the light and dark periods.

In the previous study, the disruption of *BPC3* would partially rescue phenotypic defects in *bpc1-1 bpc2* (*bpc1,2*) and *bpc1-1 bpc2 bpc4 bpc6* (*bpc1,2,4,6*) mutants (Monfared et al., 2011), indicating *BPC3* and the other BPC members have antagonistic functions (Monfared et al., 2011). As previously reported, BPC class-I mutant combinations were defective in vegetative growth (Monfared et al., 2011). The *bpc1-1 bpc2* (*bpc1,2*) double mutant was decreased in plant size, which was partially rescued in *bpc1-1 bpc2 bpc3-1* (*bpc1,2,3*), and more reduced in *bpc1,2,4,6* (Figure 1A; Monfared et al., 2011), suggesting that BPC3 impeded the growth of *bpc1,2*. We further profiled the circadian growth of *bpc* mutants to test if BPCs were involved in rhythmic growth regulation. With the contour of the rosette area across day–night cycles (Figure 1B), the wild type exhibited rhythmic changes in the expansion rate, peaking at night under the day–night process (Figure 1C). The expansion rate of the rosette area at night was inhibited in *bpc1,2* (Figure 1C), indicating that *BPC1* and *BPC2* were required for the growth control. A partial recovery of expansion accelerating before dusk was detected in *bpc1,2,3*, though the reduced expansion rate in *bpc1,2* was not fully rescued in *bpc1,2,3* (Figure 1C). This was consistent with the partial rescue of plant size in *bpc1,2,3* (Figure 1A). The rosette expansion rate was further impeded in *bpc1,2,4,6* (Figure 1D). Coincidently, the extent of inhibited growth observed in *bpc1,2* and *bpc1,2,4,6* was anticorrelated with the number of *BPC* genes in plants carrying the wild-type *BPC3*.

### BPC Members Are Antagonized Mutually in *BPC* Transcriptions

**FIGURE 2:**
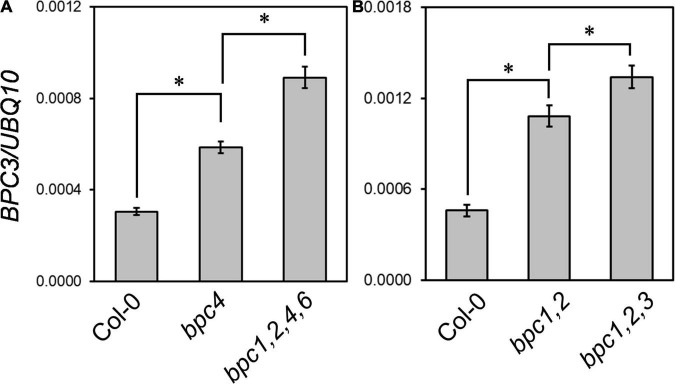
BPC family members antagonize the transcript level of *BPC3.* The expression levels of *BPC3* were determined in 18-day-old plants of Col-0, *bpc4*, *bpc1,2,4,6* **(A)**, Col-0, *bpc1,2*, and *bpc1,2,3* **(B)** by using qRT-PCR analyses with the amplicon “b” of *BPC3* shown in Figure 3A. Data are mean ± S.E. (*n* = 3 technical replicates, one independent biological replicate was presented in Supplementary Figure 1). Asterisks indicate *BPC3* transcript levels of different genetic backgrounds were significantly different (Student’s *t-*test; *P < 0.01).

It has been shown the promoter activity of *BPC3* is low in vegetative tissues where the other *BPCs* are expressed (Monfared et al., 2011), and the transcript level of *BPC3* is elevated in *bpc1,2,3,4,6* mutant roots (Shanks et al., 2018). These together implied BPC1, BPC2, BPC4, BPC6, and BPC3 itself could repress the expression of *BPC3*. We inspected if the transcript level of *BPC3* would be altered in *bpc4* and *bpc1,2,4,6* mutant plants in vegetative tissues aboveground. The transcript level of *BPC3* was increased in *bpc4* and further enhanced in the quadruple *bpc1,2,4,6* mutant (Figure 2A and Supplementary Figure 1A). Moreover, such antagonization of *BPC3* was also conducted by *BPC3* itself. The expression of *BPC3* was increased in *bpc1,2* (Figure 2B and Supplementary Figure 1B); once combined with the nonsense *bpc3-1* mutation, the transcript level of *bpc3-1* allele was further enhanced in *bpc1,2,3* (Figure 2B and Supplementary Figure 1B). This indicated that BPC3 was involved in transcriptional repression of *BPC3* itself. The antagonistic function of BPC members on *BPC3* expression showed a dosage dependence under the vegetative phase (Figure 2 and Supplementary Figure 1). Taken together with the circadian growth results, BPC members were involved in plant growth promotion, at least if not all, *via* antagonizing the expression of *BPC3*.

**FIGURE 3:**
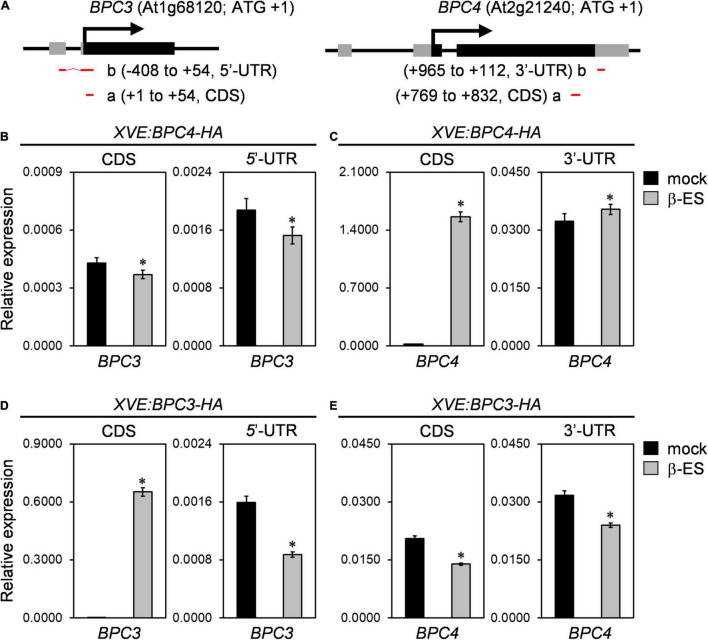
BPC members are mutually antagonized. **(A)** Diagrams of gene structures of *BPC3* (left panel) and *BPC4* (right panel). Translational start and exons are marked with arrows and boxes. Black and gray boxes illustrate the coding region sequence (CDS) and untranslated regions (UTR). Red horizontal bars “a” and “b” illustrate amplicons of qPCR. Numbers denoted in parentheses are positioned relatively to translation start site + 1 for amplicons at CDS or UTR. The illustrated *BPC3* 5′-UTR of the gene structure is revealed by the EST clone “M44A7” but not shown in the gene model of TAIR10. **(B–E)** The inducible lines of *XVE:BPC4-HA* **(B,C)** and *XVE:BPC3-HA* **(D,E)** were treated with 0 (mock) or 50 μmM 17-β-estradiol (β-ES) for 24 h and harvested every 3 h for the next day. The transcription levels of *BPC4* **(C,E)** and *BPC3* **(B,D)** relative to that of *UBQ10* were analyzed by qRT-PCRs with amplicons located at the indicated CDS or UTR. Data are mean ± S.E. (*n* = 27, each data includes 3 technical repeats of 9 biological replicates collected every 3 h across the second day after induction; corresponding individual time points and an independent biological replicate are presented in Supplementary Figure 5). Asterisks indicate transcript levels were significantly changed by β-ES treatments (Student’s *t-*test; *P < 0.05).

DNA affinity purification and sequencing (DAP-seq; O’Malley et al., 2016) has shown that BPC4 and BPC1 potentially bind to the upstream region of the BPC3 coding region (Supplementary Figure 2A). In the transcriptomic analyses of a public database (Winter et al., 2007), the transcript of *BPC4* is most abundant compared with those of functional class I and II BPC members across most developmental conditions (Supplementary Figure 3). We applied the XVE chemical system (Zuo et al., 2006) for *BPC3* and *BPC4* coding DNA sequences (CDS) induction (*XVE:BPC3-HA* and *XVE:BPC4-HA*) to examine their antagonistic functions on each other under the wild-type background (CDS, Figure 3A). The transgenic plants of *XVE:BPC3-HA* and *XVE:BPC4-HA* were treated with a series of 17-β-estradiol concentrations for 1 day, and the protein expression was determined (Supplementary Figure 4). To obtain an overview of the BPC3 and BPC4 effects on *BPC3/BPC4* expression, we profiled the transcript levels of *BPC3/BPC4* every 3 h spanning 24 h after 1-day induction.

The induction of BPC4-HA overexpression slightly compromised the overall level of *BPC3* transcript level at both CDS and 5′-UTR (Figure 3B and Supplementary Figures 5A,B). Notably, the *BPC3* decrease under BPC4-HA induction was not tremendous. This could be because the BPC redundancy (Monfared et al., 2011; Shanks et al., 2018) in the transgenic lines is functional. Due to that DAP-seq database shows that the *BPC4* locus is a potential target of BPC1 (Supplementary Figure 2B); we also examined if BPC4 regulated *BPC4* expression. Because the induction of transgenic BPC4-HA would mask the CDS of the endogenous *BPC4* profile, we analyzed the endogenous 3′-UTR of the *BPC4* transcript, which was not included in the *XVE:BPC4-HA* construct. The transcript level of the endogenous *BPC4* affected by BPC4-HA induction was minor (Figure 3C and Supplementary Figures 5C,D).

The increase in *BPC3* transcript level in *bpc1,2,3* (Figure 2B and Supplementary Figure 1B) and *bpc1,2,3,4,6* (Shanks et al., 2018) indicated that *BPC3* could antagonize its expression. We next tested if BPC3 autoregulated *BPC3*. Upon the induction of BPC3-HA, the endogenous *BPC3* transcript level was assessed by the amplicon of 5′-UTR. The endogenous *BPC3* was indeed repressed by BPC3-HA (Figure 3D and Supplementary Figures 5E,F), indicating that *BPC3* expression was moderated *via* autoregulatory machinery. Interestingly, the transcript level of *BPC4* was also repressed by BPC3-HA (Figure 3E and Supplementary Figures 5G,H), indicating that *BPC3* and *BPC4* were mutually antagonistic. The downregulation of the endogenous *BPC3* 5′-UTR by BPC3-HA was more substantial than that by BPC4-HA (Figures 3B,D and Supplementary Figures 5A,B,E,F). This suggested that BPC3 was a more stringent repressor than BPC4 in repressing *BPC3*.

### The Oscillation of the Circadian Clock Is Hampered in *bpc1,2,4,6*

**FIGURE 4:**
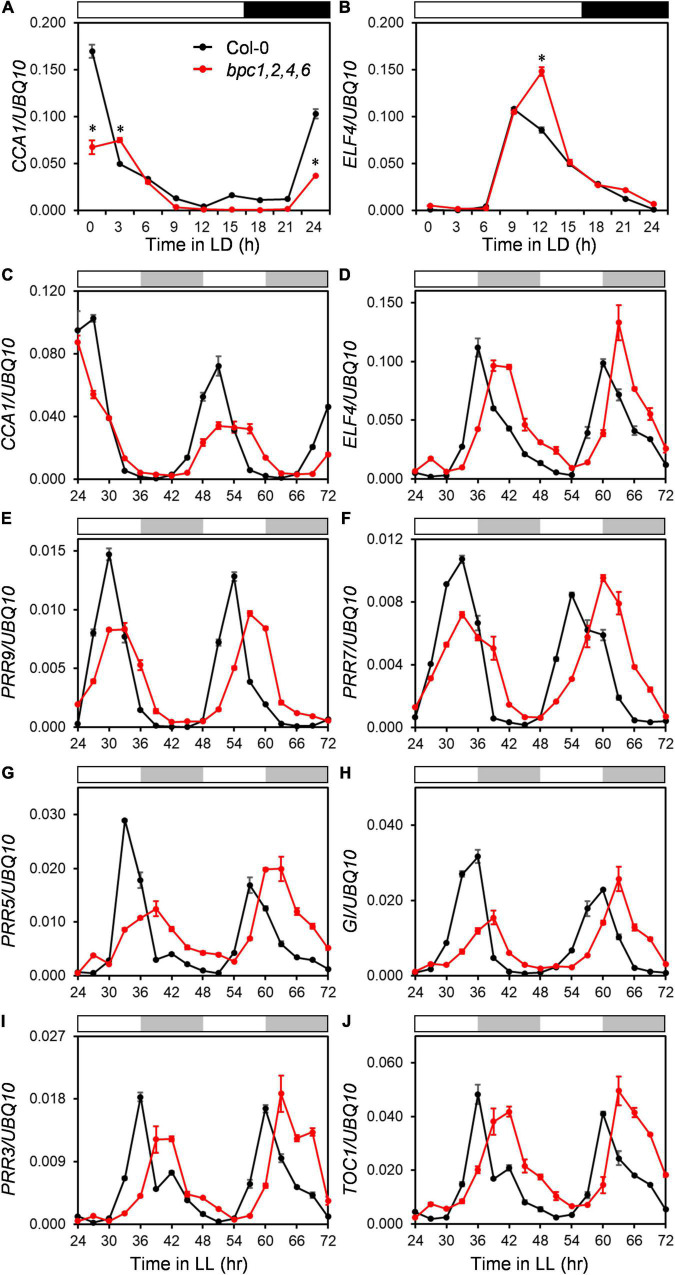
The expression of clock genes are phase-delayed in the *bpc1-1 bpc2 bpc4 bpc6* mutant. **(A,B)** Eighteen-day-old wild-type (Col-0) and *bpc1-1 bpc2 bpc4 bpc6* (*bpc1,2,4,6*) plants grown under long day (16-h light/8-h dark) were harvested at indicated ZT for profiling circadian clock representative morning gene *CCA1* **(A)** and evening gene *ELF4* **(B)**. qRT-PCR analyses were conducted, data are mean ± S.E. (*n* = 3 technical replicates). Asterisks indicate *CCA1* transcript levels were significantly altered in mutants (Student’s *t-*test; *P < 0.01). **(C–J)** Eighteen-day-old plants grown under midday (12-h light/12-h dark) were transferred to the constant light (LL) and harvested at 3-h intervals from LL24h to LL72h for *CCA1* **(C)**, *ELF4* **(D)**, *PRR9* **(E)**, *PRR7* **(F)**, *PRR5* **(G)**, *GI* **(H)**, *PRR3* **(I)**, and *TOC1* **(J)** profiling by qRT-PCR analyses. Data are mean ± S.E. (*n* = 3 technical replicates; one independent biological replicate was presented in Supplementary Figure 6). White, black, and gray bars denote the light, dark, subjective light, and subjective darkness, respectively.

**FIGURE 5:**
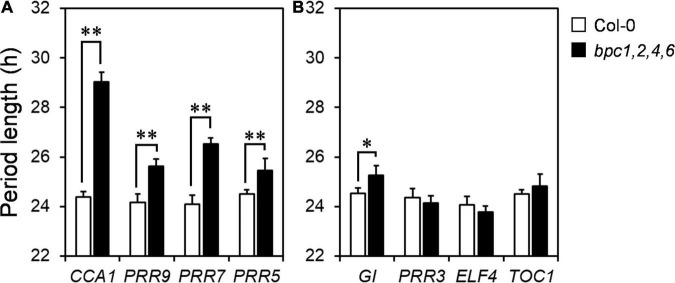
Clock genes are phase delayed in *bpc1-1 bpc2 bpc4 bpc6.* Period lengths of genes phased before **(A)** and after evening **(B)** under constant light were calculated by using MFourFit deposited at BioDare2 (Zielinski et al., 2014; https://biodare2.ed.ac.uk/). Data are mean ± S.E. (*n* = 6, the data include three technical repeats of two independent biological replicates). Asterisks indicate period length was significantly delayed in *bpc1,2,4,6* mutant (Student’s *t-*test; ^**^P < 0.01, *P < 0.05).

Since the diel growth of *bpc* mutants was impeded with lower amplitudes under day–night cycles, we next tested if the circadian clock was affected in the *bpc1,2,4,6* mutant. Two representative morning and evening genes, *CCA1* and *ELF4*, were first examined under the day–night cycle. The expression peaks of *CCA1* and *ELF4* were lately shifted by approximately 3 h (Figures 4A,B and Supplementary Figures 6A,B). The diel changes of *CCA1* and *ELF4* suggest that the oscillation of the circadian clock could be altered in the quadruple mutant. To clarify this, we characterized the clock oscillation by profiling multiple clock genes in the mutant under the free-running condition. The expression phases of *CCA1* and *ELF4* were indeed delayed in *bpc1,2,4,6* (Figures 4C,D and Supplementary Figures 6C,D). In addition to *CCA1* and *ELF4*, genes consecutively phased spanning from morning to night, including *PRR9*, *PRR7*, *PRR5*, *GI*, *PRR3*, and *TOC1*, were phase-delayed in *bpc1,2,4,6* (Figures 4E–J and Supplementary Figures 6E–J). The period length of the circadian clock in the mutant was further analyzed by using the mFourfit method in the BioDare system (Zielinski et al., 2014). Period lengths of genes phased before subjective dusk, including *CCA1*, *PRR9*, *PRR7*, and *PRR5*, were prolonged (Figure 5A). However, among genes phased after subjective dusk, *GI* was the only gene prolonging period length. Period lengths of *PRR3*, *ELF4*, and *TOC1* in *bpc1,2,4,6* were not significantly changed (Figure 5B). This revealed that BPCs functioned on the circadian clock in a gene-specific manner.

### BPCs Are Involved in Clock Regulation

**FIGURE 6:**
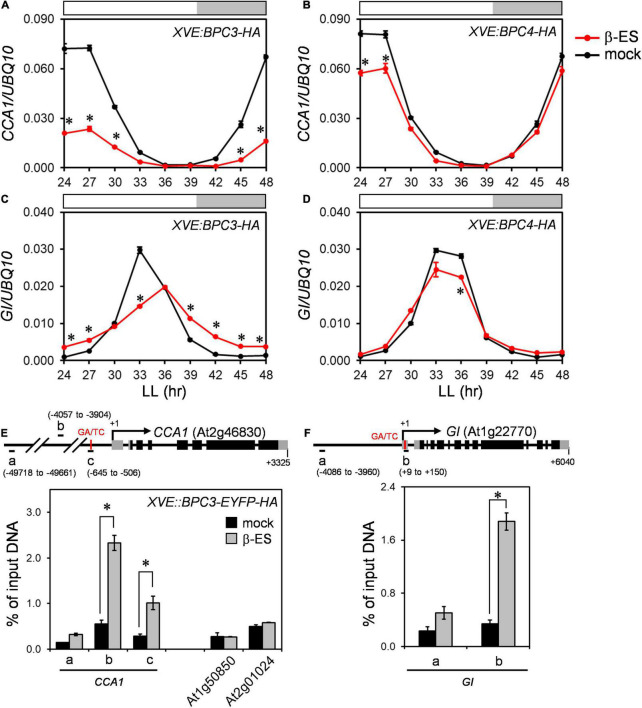
*BPC3* is upregulated in the *bpc1,2,4,6* and is a negative regulator for *CCA1.* **(A–D)** Twelve-day-old plants of *XVE:BPC3-HA* and *XVE:BPC4-HA* transgenic lines were treated with 0 (mock) or 50 μM β-estradiol (β-ES) for 24 h when released to LL and harvested at indicated times for profiling *CCA1* **(A,B)** and *GI* **(C,D)** by qRT-PCR analyses. Data are means ± S.E. (*n* = 3 technical replicates; one independent biological replicate was presented in Supplementary Figure 7). Asterisks indicate transcript levels were significantly changed by the β-ES treatment (Student’s *t-*test; *P < 0.05). **(E)** BPC3 was associated with the *CCA1* promoter *in vivo*. Leaves of 22-day-old *XVE:BPC3-EYFP-HA* transgenic plants grown under LD were treated with 0 (mock) or 50 μM β-estradiol at ZT9 for one day and fixed to conduct ChIP-qPCR analyses by using an anti-HA antibody. The diagram shows the translation start site and exons of *CCA1* gene structure and upstream region. Gray and black boxes represent untranslated and coding regions, respectively. The amplicons “a,” “b,” and “c” for ChIP-qPCR are indicated by horizontal black bars. Numbers indicate the positions relative to the transcriptional start site + 1 of *CCA1*. Data are mean ± S.E. (*n* = 3). Transposable elements At1g50850 and At2g01024 were used as negative controls. Asterisks indicate that amplicons were at least three-fold enriched by the BPC3-EYFP-HA induction significantly (Student’s *t-*test; *P < 0.01). **(F)** Chromatin immunoprecipitation qPCR was conducted as described in panel **(E)**. The diagram shows *GI* gene structure with the amplicons “a” and “b” for ChIP-qPCR assays. Data are mean ± S.E. (*n* = 3). The asterisk indicates that the amplicon was significantly enriched upon the BPC3-EYFP-HA induction (Student’s *t-*test; *P < 0.001). An independent biological replicate conducting the GA/TC association tests was shown in Supplementary Figure 7F.

One possibility that phase-delayed in *bpc1,2,4,6* could be due to increased *BPC3* expression, suggesting that *BPC3* was a repressor for clock regulation. We tested if *CCA1* could be repressed transcriptionally by BPC3. The overexpression of BPC3-HA was induced, and RNA samples were harvested at 3-h intervals across 24–48 h induction time under the free-running condition. The expression of *CCA1* was significantly compromised under the BPC3-HA induction (Figure 6A and Supplementary Figure 7A), indicating that BPC3 was indeed capable of *CCA1* repression. The repression of *CCA1* under BPC3-HA induction was consistent with the anticorrelation between *BPC3* and *CCA1* transcript levels in *bpc1,2,4,6*. The decrease of *CCA1* expression in *bpc1,2,4,6* was also possibly due to a lack of activation by BPCs except for BPC3. We introduced the overexpression of BPC4-HA to test this hypothesis. Instead of being activated, the expression of *CCA1* was slightly repressed under the induction of BPC4-HA overexpression (Figure 6B and Supplementary Figure 7B). This indicates that the role of BPC4 in *CCA1* mediation should be negative instead of positive. Such a BPC4-repressive effect was consistent with its overlapping role with BPC6 in class II BPC-dependent recruitment of polycomb-repressive complexes (PRCs) for transcription repression (Hecker et al., 2015).

Consistent with weak repression conducted by BPC4-HA on *BPC3*, we again observed that the induction of BPC4-HA overexpression had just mildly repressed *CCA1*. Perhaps, endogenous BPC4 might merely leave a subtle extent for transgenic BPC4-HA to repress *CCA1*. Therefore, the induction of BPC4-HA could not further repress *CCA1* drastically. If this was the case, lacking endogenous BPC4 should increase the expression level of *CCA1*. We examined the *CCA1* expression profile in the *bpc4* mutant. The expression of the *CCA1* level in *bpc4* was compromised slightly (Supplementary Figure 7C). This indicated that the endogenous BPC4 unlikely repressed *CCA1* significantly. Rationally, the decrease of *CCA1* in *bpc4* might be due to the ectopic expression of *BPC3* in the *bpc4* mutant (Figure 2A and Supplementary Figure 1A). Taken together, BPC4 was a less stringent repressor than BPC3 for *CCA1* regulation.

The low expression of *CCA1* shortens the clock period (Lu et al., 2009). However, the repression of *CCA1* in *bpc1,2,4,6* prolonged the period (Figures 4A,C, 5A and Supplementary Figures 6A,C). This might be because *CCA1* was not the only clock gene repressed by BPC3. The period length analysis showed that the period of *GI* expression was a dusk gene lengthened in *bpc1,2,4,6* (Figures 4H, 5B and Supplementary Figure 6H). We next examined the *GI* expression under the *BPC3-HA* induction and found that the peak of *GI* was indeed compromised and phase-delayed by BPC3-HA (Figure 6C and Supplementary Figure 7D). The induction of BPC4-HA overexpression only slightly compromised the *GI* peak (Figure 6D and Supplementary Figure 7E). Consistently with the regulation of *CCA1*, BPC3 and BPC4 would repress *GI* with different stringencies, indicating that BPCs regulate the circadian clock *via* multiple genes. Moreover, the simultaneously repressed *GI* would lengthen the circadian clock period (Fowler et al., 1999). This may best explain the long period caused by the ectopic BPC3 in *bpc1,2,4,6* and by BPC3-HA induction while *CCA1* was repressed.

### BPCs Are Involved in *CCA1* and *GI* Regulation

A cis-element sharing pattern of the “AGARRGARRRAGADR” element of the plant-specific GAGA-motif has been identified in the region (–716 to –704) upstream of the *CCA1* transcriptional start site (Gendron et al., 2012). Besides, another potential GAGA-motif comprising a quintuple repeat of GA/TC dinucleotide can be found in the region 122 bp (5 × GA, –581 to –570) next to the above GAGA-motif. We asked if BPC3 directly targeted *CCA1* for clock regulation *in vivo*. An inducible construct of EYFP-HA-tagged BPC3 (*BPC3-EYFP-HA*) was generated to track the expression of BPC3 in cells. Upon the induction, the BPC3-EYFP-HA protein was detected with the predicted molecular weight in transgenic plants (Supplementary Figure 8A). The BPC3-EYFP-HA showed a punctate pattern across leaf blades (Supplementary Figure 8B), constituted by the nucleus locating signal of BPC3-EYFP-HA under the cellular scope (Supplementary Figure 8C). Chromatins targeted by BPC3-EYFP-HA were then immunoprecipitated with anti-HA antibody and used for the qPCR analysis. The amplicon “c” (–645 to –506 upstream of the transcription start site + 1 of *CCA1*) comprising the annotated quintuple GA/TC-repeat was enriched only when BPC3-EYFP-HA was induced (Figure 6E and Supplementary Figure 8D). BPC3 did not significantly associate with a remote region upstream of the *CCA1* promoter (amplicon “a,” –49718 to –49661, Figure 6E), nor with two transposable element genes, At1g50850 and At2g01024 (Figure 6E). These together indicated that BPC3 specifically targeted the *CCA1* promoter *in vivo*.

Strikingly, amplicon “b” (–4057 to –3904), which is 3.3-kb upstream from the putative GAGA-motif, was also highly enriched in the BPC3 associated chromatin (Figure 6E), 1 kb from the nearest pentamerous GA/TC-dinucleotide (–5077 to –5068). The DNA affinity purification and sequencing (DAP-seq; O’Malley et al., 2016) identified amplicon “b” showing DAP-seq binding signals by BPC4 (Supplementary Figure 8E), suggesting the amplicon includes *bona fide* BPC target sequences. Our results indicated that BPC3 negatively regulated *CCA1 via* direct promoter targeting. A GAGA-motif can be identified at the 5′-UTR of *GI*, which was also targeted by BPC4 in public DAP-seq data (Supplementary Figure 8F). The results of our ChIP-qPCR assays also showed that BPC3 directly targeted a region harboring the GAGA-motif located at 5′-UTR of *GI* but not a 4-kb upstream region (Figure 6F and Supplementary Figure 8D). Collectively, *CCA1* and *GI* were targeted by BPC3.

### The Induction of BPC3 Overexpression Affects a Subset of *BPCs*

**FIGURE 7:**
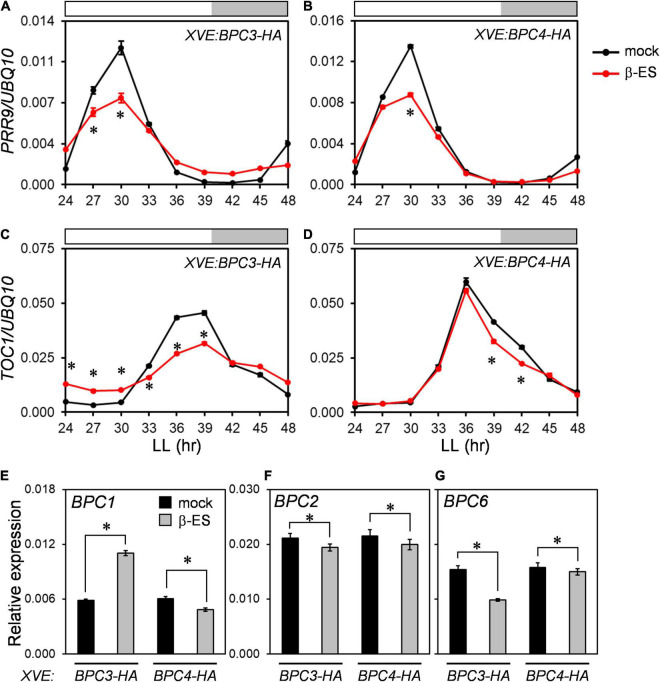
*BPCs* negatively regulated circadian clock components. **(A–D)** qRT-PCR analyses for expression profiles of *PRR9* **(A,B)** and *TOC1* **(C,D)** under the induction of *BPC3-HA* **(A,C)** and *BPC4-HA* **(B,D)** transgenic lines as described in Figures 6A–D. **(E–G)** Expression levels of *BPC1* **(E)**, *BPC2* **(F)**, and *BPC6* **(G)** were profiled under *BPC3-HA* or *BPC4-HA* induction. Asterisks indicate the transcript levels were significantly changed by the β-ES treatment (Student’s *t-*test; *P < 0.05). Data are mean ± S.E. (*n* = 27, data collected as described in Figure 3; an independent biological replicate and corresponding individual time points are presented in Supplementary Figure 9).

Even though the phases and amplitudes of *CCA1* and *GI* were delayed and compromised in *bpc1,2,4,6* and under BPC3-HA overexpression (Figures 4, 6), the expression profiles of *PRR9* and *TOC1* were repressed by BPC3-HA or BPC4-HA induction without significantly delaying expression peaks (Figures 7A–D and Supplementary Figures 9A–D). The induction of BPC3-HA overexpression under a wild-type background did not fully replicate clock defects shown in the *bpc1,2,4,6* quadruple mutant. Therefore, the ectopic expression of BPC3 was not sufficient to alter the clock oscillation, and simultaneously lacking *BPC1*, *BPC2*, *BPC4*, and *BPC6* was also required.

As the endogenous *BPC4* was repressed under the BPC3-HA induction (Figure 3E and Supplementary Figures 5G,H), we further analyzed transcript levels of *BPC1*, *BPC2*, and *BPC6* under the BPC3-HA induction to assess if other BPC members were affected. Contradictory with the *BPC4* repression by BPC3-HA, the transcript level of *BPC1* was nearly two-fold upregulated by the BPC3-HA induction (Figure 7E and Supplementary Figure 9E). *BPC2* was moderated, and *BPC6* was inhibited by BPC3-HA, respectively (Figures 7F–G and Supplementary Figures 9F–G). Again, BPC4-HA induction slightly inhibited the expression of *BPC1*, *BPC2*, and *BPC6* (Figures 7E–G and Supplementary Figures 9E–G). BPC4-HA can be a mild repressor of *BPC1*, *BPC2*, and *BPC6*. These findings indicated that BPC3 could regulate a subset of BPCs and supported that *BPC* members, including *BPC3*, co-regulated the circadian clock.

The public DAP-seq data revealed that *CCA1* and *GI* are potential targets of BPC binding (Supplementary Figures 8E,F). We next examined if *CCA1* and *GI* were also targeted by BPC1, which was upregulated by the BPC3-HA induction. We conducted ChIP-qPCR assays within the *BPC1-EYFP-HA* transgenic lines. ChIP-qPCR demonstrated a clear association of BPC1 with the upstream region (–645 to –506 bp) of *CCA1* and the 5′-UTR of *GI* (Supplementary Figure 9H), which implied the *bona fide* regulation of the circadian clock by multiple BPCs. Therefore, the BPC3-HA induction has interfered with the network constituted by BPCs in clock regulation.

### BPC3 Overexpression Causes the Disorder of Leaf Development

**FIGURE 8:**
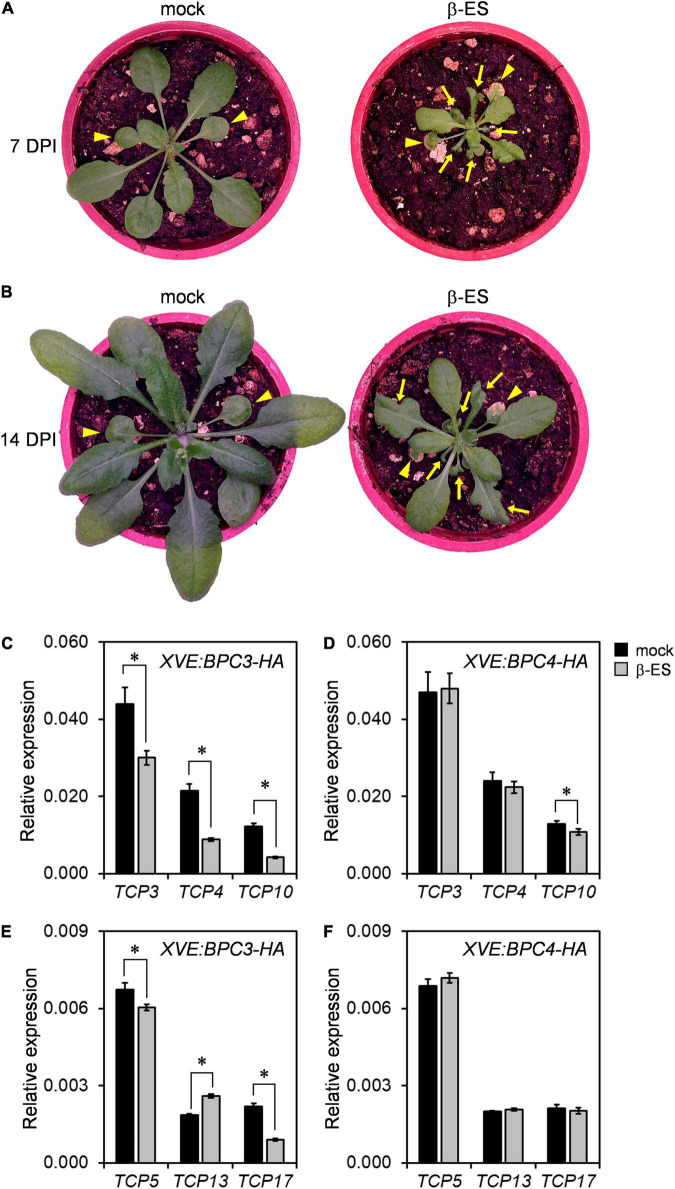
The ectopic expression of BPC3 impedes leaf development and growth. **(A,B)** Plants of 14-day-old *XVE:BPC3-EYFP-HA* were imaged on the 7th **(A)** and 14th days **(B)** post induction (DPI) by 0 or 50 μmM β-estradiol (β-ES). Arrowheads indicate the first two true leaves. Arrows indicate the impeded growth and edge formation of younger leaves of the transgenic plants. **(C,D)** Expressions of *TCP3*, *TCP4*, and *TCP10* were analyzed by qRT-PCR under the induction of *XVE:BPC3-HA* **(C)** and *XVE:BPC4-HA* **(D)**. **(E,F)** The expressions of *TPC5*, *TCP13*, and *TCP17* were analyzed under indicated inductions. The expressions of the indicated *TCPs* were relative to that of *UBQ10*. Data are mean ± S.E. (*n* = 27, data collected as described in Figure 3; an independent biological replicate is presented in Supplementary Figures 10A–D, individual time points are shown in Supplementary Figure 11). Asterisks indicate expressions significantly changed by 50 μmM β-ES treatment (Student’s *t-*test; *P < 0.05).

The leaf morphology of the *bpc1,2,4,6* mutant is shrunken and curled (Monfared et al., 2011). This suggests that the ectopic *BPC3* overexpression during the vegetative phase would impede leaf development. We induced *BPC3-EYFP-HA* overexpression in transgenic plants by spreading 17-β-estradiol onto tissues aboveground of transgenic lines at the stage of 14-day-old. After 7 days of BPC3-EYFP-HA induction, the size of 17-β-estradiol-treated plants was decreased, and the blades of juvenile leaves were heavily curled (Figure 8A). This suggested that the ectopic BPC3 expression hampered the edge formation under leaf expansion and decreased the rosette expansion.

While the effect of the inducer declined with time, leaf growth was gradually recovered (Figure 8B). The residue parts of growth-impeded and edge-curled leaves were enlarged, and later adult leaves with normal edge formation were generated after 14 days of the induction (Figure 8B). The alternation in leaf morphology by the induction of BPC3 overexpression or shown by *bpc1,2,4,6* (Monfared et al., 2011) suggested that the repression of *BPC3* by BPCs was crucial for sustaining leaf growth and development. TEOSINTE BRANCHED1-CYCLOIDEA-PCFs (TCPs), a family of transcription factors controlling leaf curvature (Nath et al., 2003; Koyama et al., 2017; Jiang et al., 2018), were further examined in the inducible *XVE:BPC3-HA* and *XVE:BPC4-HA* lines. *TCP3*, *TCP4*, and *TCP10* are essential *TCP* members targeted by miR319 and required for leaf edge development (Koyama et al., 2017). Their transcript levels were significantly reduced under the induction of *BPC3-HA* (Figure 8C and Supplementary Figures 10A, 11) but marginally reduced (less than 25% reduction) by *BPC4-HA* (Figure 8D and Supplementary Figures 10B, 11). We also tested the expression of *TCP5*, *TCP13*, and *TCP17*, family members not targeted by miR319 (Koyama et al., 2017). *TCP5* was moderated, *TCP13* and *TCP17* were, respectively, increased and repressed by the BPC3-HA (Figures 8E–F and Supplementary Figures 10C,D, 11) and moderated by the BPC4-HA in transgenic plants upon the inducer (Figures 8E–F and Supplementary Figures 10C,D, 11). This revealed that BPC3 was involved in leaf morphology control, mainly *via* the repression of *TCP3/4/10/17*.

It was noticed that the first two leaves were precociously yellowing under BPC3-EYFP-HA induction (Figure 8A), likely the leaf senescence was triggered. This implied that *BPC3* was involved in the leaf senescence. We examined the expression of *BIFUNCTIONAL NUCLEASE 1* (*BFN1*), the senescence-associated nuclease I gene (Peìrez-Amador et al., 2000). The BPC3-HA induction indeed enhanced the expression of *BFN1*, which was slightly repressed by the BPC4-HA induction (Supplementary Figures 10E, 11). This supports that the yellowing leaf can be due to precocious senescence. However, this could contrast with the repression of *TCPs* by BPC3-HA induction (Figure 8 and Supplementary Figures 10A–D), since TCPs play roles in stimulating leaf senescence (Koyama et al., 2017). This implied that the induction of BPC3-HA might trigger the leaf senescence pathway *via* an independent path of *TCPs* examined in this study.

## Discussion

**FIGURE 9:**
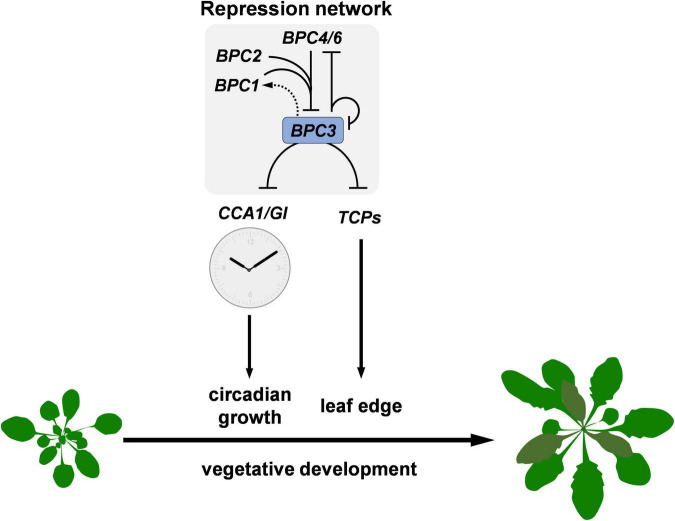
Diagram depicting the repression machinery constituted by BPC members in Arabidopsis vegetative development. Family members BPC1, BPC2, BPC3, BPC4, and BPC6 additively formed a repression network to limit *BPC3* expression during the vegetative development. The concurrent mutations on *BPC1*, *BPC2*, *BPC4*, and *BPC6* cause the relief of repression on *BPC3*. The ectopic BPC3 represses multiple clock genes including *CCA1* and *GI*, resulting in the retardation of circadian growth. Simultaneously, BPC3 impedes the formation of leaf edge *via* repressing a subset of *TCPs* essential for leaf development. The reciprocal regulations between BPC members are marked with black lines with arrows (positive) or blunt ends (negative) according to the genetic study by Monfared et al. (2011) and BPC3/4 functional assays in this study. BPC3 may activate *BPC1 via* an indirect mechanism marked as a dashed arrow.

Our study discloses the action roles of BPC3, a hidden repressor repressed by other BPCs, in harming multiple vegetative developmental processes (Figure 9). BPC family members are plant-specific transcription factors involved in numerous developmental processes, usually directly bound to GAGA motifs. BPCs regulate transcription *via* PRC1/2 complex-dependent pathways (Hecker et al., 2015; Wu et al., 2020) or by cooperating with PRCs and MADS-domain factors simultaneously to synergistically repress the target *STK* (Simonini et al., 2012; Petrella et al., 2020). The mechanism that BPCs regulate downstream genes is also applicable to tuning *BPC* expression. The transcript level of *BPC3* is increased in *bpc4*, *bpc1,2*, *bpc1,2,3*, *bpc1,2,4,6* (Figure 2 and Supplementary Figure 1; Monfared et al., 2011) and *bpc1,2,3,4,6* mutants (Monfared et al., 2011; Shanks et al., 2018), suggesting that other BPCs and BPC3 itself have functioned on the repression of *BPC3*. Our BPC3 induction tests also revealed that BPC3 is a repressor of *BPC3* (Figure 3D and Supplementary Figures 5E,F), unraveling a feedback loop in which BPC3 auto represses its transcription with other BPCs (Figure 9). *BPC3* is not the only family member regulated by BPCs. *BPC1*, *BPC2*, *BPC4*, and *BPC6* are targeted by BPC6 (Shanks et al., 2018), of which transcription level is decreased under the induction of BPC3 overexpression (Figure 7G and Supplementary Figure 9G). In addition to the repression, we discover that BPC3 is also positively involved in the *BPC1* regulation (Figure 7E and Supplementary Figure 9E), which might be potentially upregulated by BPC6 (Shanks et al., 2018). Collectively, BPC family members, including BPC3, are involved in constituting a BPC-repressive network for transcription (Figure 9).

Upon the defects in higher-order mutants composed of concurrent mutations of *BPC3* and other *BPCs*, *BPC3* and other *BPCs* are proved redundantly functioning on meristem size maintenance and root cytokinin responses (Simonini and Kater, 2014; Shanks et al., 2018). However, the transcript level of *BPC3* is immensely lower than that of other BPCs in seedlings across the vegetative phase and is only increased if one or multiple *BPCs* are compromised (Monfared et al., 2011; Mu et al., 2017; Shanks et al., 2018). Consequently, the function of BPC3 could be redundant or might merely moderate developmental processes with other BPCs during the vegetative phase. However, the double and higher-order *bpc* mutants harbored wild-type *BPC3* also show multiple developmental defects (Figure 1; Monfared et al., 2011; Hecker et al., 2015; Wu et al., 2020). Given that developmental defects shown by *bpc1,2* and *bpc1,2,4,6* are partially rescued, respectively, in the triple mutant *bpc1,2,3* (Figure 1) and quintuple mutant *bpc1,2,3,4,6* (Monfared et al., 2011), one crucial function of *BPCs* for the development can be antagonizing *BPC3*. This study revealed that masking BPC3 activity by the BPC-repressive network is vital for vegetative growth. The induction of BPC3 overexpression causes phenotypes resembling the defected traits shown by *bpc1,2,4,6*. Once the BPC3 overexpression (Figure 3 and Supplementary Figure 5) or *bpc* mutation (Figure 2 and Supplementary Figure 1) interferes with the repression of *BPC3*, the essential genes of the circadian clock and leaf edge formation are concurrently repressed (Figures 4–8 and Supplementary Figures 6, 7, 9–11). The circadian growth and leaf development, later on, are impeded due to the defects of the controlling molecular mechanisms (Figure 8 and Supplementary Figures 10, 11).

All the circadian clock genes that we examined are phase-delayed plausibly because the genes are interlocked in a complex network of the circadian system (Figure 4 and Supplementary Figure 6). We found that the BPC-repressive network functions on the circadian clock by directly targeting *CCA1* and *GI*, at least *via* BPC1 and BPC3 (Figures 6, 7). Moreover, BPCs might also cooperate with circadian clock components. The *CCA1* promoter region targeted in our BPC3- and BPC1-associated ChIP-qPCR assays encompassing a GAGA *cis*-element “AAGGAGGAAGAAG” (Figures 6E,F and Supplementary Figure 9H), which is concurrently targeted by the direct repressor TOC1 of *CCA1* (Gendron et al., 2012; Huang et al., 2012; Pokhilko et al., 2012). Given that the induction of TOC1 would upregulate a subset of genes sharing the sequence pattern “AGARRGARRRAGADR” possessing the putative GAGA motifs at the 500-bp promoter region (Gendron et al., 2012). It is likely TOC1 and BPCs co-regulate a group of targets.

The induction of BPC4 did not repress the expression of *CCA1* and *GI* as BPC3 did (Figure 6 and Supplementary Figure 7), suggesting that BPC3 and BPC4 have different capabilities for downstream gene regulation. The other regulations conducted by BPC3 and BPC4 on *BPCs* and *TCPs* are also differential. Most *BPCs* and *TCPs* repressed by BPC3 were merely moderated by BPC4 (Figures 3, 7, 8 and Supplementary Figures 5, 9–11). However, the weak repression by BPC4 could be because the downstream genes we examined are not preferential targets of BPC4. The overexpression of BPC4 constitutively represses the transcript level of *ABI4* in Arabidopsis roots during the early stage of seedling growth (Mu et al., 2017). Potentially, different preferences for target regulations among BPC members are broadly exerted in other tissues at different developmental stages.

Our ChIP-qPCR results demonstrate that BPC1 and BPC3 bind to the GAGA targets of BPC4 identified in the public DAP-seq database (Supplementary Figures 8E,F). This suggests that BPC domains at the C-terminus of BPC members possess a DNA binding generally for the GAGA motif recognition (Santi et al., 2003; Monfared et al., 2011; Theune et al., 2019). It has been shown that a functional motif required for the dimerization and interaction with LHP1 of the PRC1 components is shared by the subclass members with BPC4 but not by members with BPC1 and BPC3 (Wanke et al., 2011; Hecker et al., 2015; Theune et al., 2019). Conceivably, variations of target regulations can be contributed to by the different consensus motifs arranged in the N-terminus outside the conserved BPC domains of BPC3 and BPC4 (Theune et al., 2019). Whether N-terminus motifs determine the target selection or modulate the repressive capacities of different class BPCs is of great interest and demands future studies.

Although BPC3 plays a minor role or function only under conditions that remain unidentified during vegetative development, BPC3 can interfere with multiple processes that are concurrently regulated by other BPCs. We uncover that *BPC3* is a hidden transcriptional repressor which has no assessable function for regulating plant development; nonetheless, the overdose of BPC3 simultaneously represses a subset of developmental genes. Otherwise, BPC4, one of the mainly expressed BPCs in Arabidopsis vegetative tissues, is a relatively modest repressor. BPC4 represses *BPC3* and tunes the developmental genes by collaborating with other BPCs in an additive way. We conclude that keeping the low profile of *BPC3* expression is a crucial function of BPCs. Our study sheds light on adverse transcriptional impacts limited by the BPC-repressive network of plant development.

### Accession Numbers

Gene information from this article are found in Arabidopsis Genome Initiative data library with locus identifiers: *BPC1* (At2g01930), *BPC2* (At1g14685), *BPC3* (At1g68120), *BPC4* (At2g21240), *BPC6* (At5g42520), *BFN1* (At1g11190), *CCA1* (At2g46830), *ELF4* (At2g40080), *PRR9* (At2g46790), *PRR7* (At5g02810), *PRR5* (At5g24470), *PRR3* (At5g60100), *TOC1* (At5g61380), *GI* (At1g22770), *TCP3* (At1g53230), *TCP4* (At3g15030), *TCP5* (At5g60970), *TCP10* (At2g31070), *TCP13* (At3g02150), *TCP17* (At5g08070), *UBQ10* (At4g05320).

## Data Availability Statement

The original contributions presented in the study are included in the article/Supplementary Material, further inquiries can be directed to the corresponding author.

## Author Contributions

Y-CL, P-TT, X-XH, and H-LT designed the research, analyzed the data, performed the research, and wrote the article. All authors contributed to the article and approved the submitted version.

## Conflict of Interest

The authors declare that the research was conducted in the absence of any commercial or financial relationships that could be construed as a potential conflict of interest.

## Publisher’s Note

All claims expressed in this article are solely those of the authors and do not necessarily represent those of their affiliated organizations, or those of the publisher, the editors and the reviewers. Any product that may be evaluated in this article, or claim that may be made by its manufacturer, is not guaranteed or endorsed by the publisher.

## References

[B1] Alabadi D. Oyama T. Yanovsky M. J. Harmon F. G. Mas P. Kay S. A. (2001). Reciprocal regulation between TOC1 and LHY/CCA1 within the Arabidopsis circadian clock. *Science* 293 880–883. 10.1126/science.1061320 11486091

[B2] Chang S. Puryear J. Cairney J. (1993). A simple and efficient method for isolating RNA from pine trees. *Plant Mol. Biol. Rep.* 11 113–116. 10.1385/MB:19:2:201

[B3] Dowson-Day M. J. Millar A. J. (1999). Circadian dysfunction causes aberrant hypocotyl elongation patterns in Arabidopsis. *Plant J.* 17 63–71. 10.1046/j.1365-313x.1999.00353.x 10069068

[B4] Engelmann W. Johnsson A. (1998). “Rhythms in organ movement,” in *Biological Rhythms and Photoperiodism in Plants*, eds Lumsden P. J. Millar A. J. (Oxford: BIOS Scientific Publishers), 35–50.

[B5] Fowler S. Lee K. Onouchi H. Samach A. Richardson K. Morris B. (1999). GIGANTEA: a circadian clock-controlled gene that regulates photoperiodic flowering in Arabidopsis and encodes a protein with several possible membrane-spanning domains. *EMBO J.* 18, 4679–4688. 10.1093/emboj/18.17.4679 10469647 PMC1171541

[B6] Gendron J. M. Pruneda-Paz J. L. Doherty C. J. Gross A. M. Kang S. E. Kay S. A. (2012). Arabidopsis circadian clock protein, TOC1, is a DNA-binding transcription factor. *Proc. Natl. Acad. Sci. U. S. A.* 109 3167–3172. 10.1073/pnas.1200355109 22315425 PMC3286946

[B7] Harmer S. L. Hogenesch J. B. Straume M. Chang H. S. Han B. Zhu T. (2000). Orchestrated transcription of key pathways in Arabidopsis by the circadian clock. *Science* 290 2110–2113. 10.1126/science.290.5499.2110 11118138

[B8] Hecker A. Brand L. H. Peter S. Simoncello N. Kilian J. Harter K. (2015). The Arabidopsis GAGA-Binding Factor BASIC PENTACYSTEINE6 Recruits the POLYCOMB-REPRESSIVE COMPLEX1 Component LIKE HETEROCHROMATIN PROTEIN1 to GAGA DNA Motifs. *Plant Physiol.* 168 1013–1024. 10.1104/pp.15.00409 26025051 PMC4741334

[B9] Huang W. Perez-Garcia P. Pokhilko A. Millar A. J. Antoshechkin I. Riechmann J. L. (2012). Mapping the core of the Arabidopsis circadian clock defines the network structure of the oscillator. *Science* 336 75–79. 10.1126/science.1219075 22403178

[B10] Jiang W. Li Z. Yao X. Zheng B. Shen W.-H. Dong A. (2018). jaw-1D: a gain-of-function mutation responsive to paramutation-like induction of epigenetic silencing. *J. Exp. Bot.* 70 459–468. 10.1093/jxb/ery365 30346598 PMC6322565

[B11] Jouve L. Greppin H. Agosti R. D. (1998). Arabidopsis thaliana floral stem elongation: evidence for an endogenous circadian rhythm. *Plant Physiol. Biochem.* 36 469–472.

[B12] Kooiker M. Airoldi C. A. Losa A. Manzotti P. S. Finzi L. Kater M. M. (2005). BASIC PENTACYSTEINE1, a GA binding protein that induces conformational changes in the regulatory region of the homeotic Arabidopsis gene SEEDSTICK. *Plant Cell* 17 722–729. 10.1105/tpc.104.030130 15722463 PMC1069694

[B13] Koyama T. Mitsuda N. Seki M. Shinozaki K. Ohme-Takagi M. (2010). TCP Transcription Factors Regulate the Activities of ASYMMETRIC LEAVES1 and miR164, as Well as the Auxin Response, during Differentiation of Leaves inArabidopsis. *Plant Cell* 22 3574–3588. 10.1105/tpc.110.075598 21119060 PMC3015130

[B14] Koyama T. Sato F. Ohme-Takagi M. (2017). Roles of miR319 and TCP Transcription Factors in Leaf Development. *Plant Physiol.* 175 874–885. 10.1104/pp.17.00732 28842549 PMC5619901

[B15] Lu S. X. Knowles S. M. Andronis C. Ong M. S. Tobin E. M. (2009). CIRCADIAN CLOCK ASSOCIATED1 and LATE ELONGATED HYPOCOTYL Function Synergistically in the Circadian Clock of Arabidopsis. *Plant Physiol.* 150 834–843. 10.1104/pp.108.133272 19218364 PMC2689956

[B16] Meister R. J. Williams L. A. Monfared M. M. Gallagher T. L. Kraft E. A. Nelson C. G. (2004). Definition and interactions of a positive regulatory element of the Arabidopsis INNER NO OUTER promoter. *Plant J.* 37 426–438. 10.1046/j.1365-313x.2003.01971.x 14731261

[B17] Monfared M. M. Simon M. K. Meister R. J. Roig-Villanova I. Kooiker M. Colombo L. (2011). Overlapping and antagonistic activities of BASIC PENTACYSTEINE genes affect a range of developmental processes in Arabidopsis. *Plant J.* 66 1020–1031. 10.1111/j.1365-313X.2011.04562.x 21435046

[B18] Mu Y. Zou M. Sun X. He B. Xu X. Liu Y. (2017). BASIC PENTACYSTEINE Proteins Repress ABSCISIC ACID INSENSITIVE4 Expression via Direct Recruitment of the Polycomb-Repressive Complex 2 in Arabidopsis Root Development. *Plant Cell Physiol.* 58 607–621. 10.1093/pcp/pcx006 28138058

[B19] Murashige T. Skoog F. (1962). A Revised Medium for Rapid Growth and Bio Assays with Tobacco Tissue Cultures. *Physiol. Plant.* 15 473–497.

[B20] Nakamichi N. Kiba T. Henriques R. Mizuno T. Chua N. H. Sakakibara H. (2010). PSEUDO-RESPONSE REGULATORS 9, 7, and 5 are transcriptional repressors in the Arabidopsis circadian clock. *Plant Cell* 22 594–605. 10.1105/tpc.109.072892 20233950 PMC2861452

[B21] Nakamichi N. Kita M. Ito S. Yamashino T. Mizuno T. (2005). PSEUDO-RESPONSE REGULATORS, PRR9, PRR7 and PRR5, together play essential roles close to the circadian clock of Arabidopsis thaliana. *Plant Cell Physiol.* 46 686–698. 10.1093/pcp/pci086 15767265

[B22] Nath U. Crawford B. C. W. Carpenter R. Coen E. (2003). Genetic Control of Surface Curvature. *Science* 299 1404–1407. 10.1126/science.1079354 12610308

[B23] O’Malley R. C. Huang S. C. Song L. Lewsey M. G. Bartlett A. Nery J. R. (2016). Cistrome and Epicistrome Features Shape the Regulatory DNA Landscape. *Cell* 166:1598. 27610578 10.1016/j.cell.2016.08.063

[B24] Palatnik J. F. Allen E. Wu X. Schommer C. Schwab R. Carrington J. C. (2003). Control of leaf morphogenesis by microRNAs. *Nature* 425 257–263. 10.1038/nature01958 12931144

[B25] Petrella R. Caselli F. Roig-Villanova I. Vignati V. Chiara M. Ezquer I. (2020). BPC transcription factors and a Polycomb Group protein confine the expression of the ovule identity gene SEEDSTICK in Arabidopsis. *Plant J.* 102 582–599. 10.1111/tpj.14673 31909505

[B26] Peìrez-Amador M. A. Abler M. L. De Rocher E. J. Thompson D. M. Van Hoof A. Lebrasseur N. D. (2000). Identification of BFN1, a Bifunctional Nuclease Induced during Leaf and Stem Senescence in Arabidopsis. *Plant Physiol.* 122 169–180. 10.1104/pp.122.1.169 10631260 PMC58855

[B27] Pokhilko A. Fernandez A. P. Edwards K. D. Southern M. M. Halliday K. J. Millar A. J. (2012). The clock gene circuit in Arabidopsis includes a repressilator with additional feedback loops. *Mol. Syst. Biol.* 8:574. 10.1038/msb.2012.6 22395476 PMC3321525

[B28] Pokhilko A. Hodge S. K. Stratford K. Knox K. Edwards K. D. Thomson A. W. (2010). Data assimilation constrains new connections and components in a complex, eukaryotic circadian clock model. *Mol. Syst. Biol.* 6:416. 10.1038/msb.2010.69 20865009 PMC2964123

[B29] Pruneda-Paz J. L. Breton G. Para A. Kay S. A. (2009). A functional genomics approach reveals CHE as a component of the Arabidopsis circadian clock. *Science* 323 1481–1485. 10.1126/science.1167206 19286557 PMC4259050

[B30] Sangwan I. O’Brian M. R. (2002). Identification of a soybean protein that interacts with GAGA element dinucleotide repeat DNA. *Plant Physiol.* 129 1788–1794. 10.1104/pp.002618 12177492 PMC166767

[B31] Santi L. Wang Y. Stile M. R. Berendzen K. Wanke D. Roig C. (2003). The GA octodinucleotide repeat binding factor BBR participates in the transcriptional regulation of the homeobox gene Bkn3. *Plant J.* 34 813–826. 10.1046/j.1365-313x.2003.01767.x 12795701

[B32] Shanks C. M. Hecker A. Cheng C. Y. Brand L. Collani S. Schmid M. (2018). Role of BASIC PENTACYSTEINE transcription factors in a subset of cytokinin signaling responses. *Plant J.* 95 458–473. 10.1111/tpj.13962 29763523

[B33] Shim J. S. Kubota A. Imaizumi T. (2017). Circadian Clock and Photoperiodic Flowering in Arabidopsis: CONSTANS Is a Hub for Signal Integration. *Plant Physiol.* 173 5–15. 10.1104/pp.16.01327 27688622 PMC5210731

[B34] Simonini S. Kater M. M. (2014). Class I BASIC PENTACYSTEINE factors regulate HOMEOBOX genes involved in meristem size maintenance. *J. Exp. Bot.* 65 1455–1465. 10.1093/jxb/eru003 24482368 PMC3967085

[B35] Simonini S. Roig-Villanova I. Gregis V. Colombo B. Colombo L. Kater M. M. (2012). Basic pentacysteine proteins mediate MADS domain complex binding to the DNA for tissue-specific expression of target genes in Arabidopsis. *Plant Cell* 24 4163–4172. 10.1105/tpc.112.103952 23054472 PMC3517243

[B36] Theune M. L. Bloss U. Brand L. H. Ladwig F. Wanke D. (2019). Phylogenetic Analyses and GAGA-Motif Binding Studies of BBR/BPC Proteins Lend to Clues in GAGA-Motif Recognition and a Regulatory Role in Brassinosteroid Signaling. *Front. Plant Sci.* 10:466. 10.3389/fpls.2019.00466 31057577 PMC6477699

[B37] Wang Y. Wu J. F. Nakamichi N. Sakakibara H. Nam H. G. Wu S. H. (2011). LIGHT-REGULATED WD1 and PSEUDO-RESPONSE REGULATOR9 form a positive feedback regulatory loop in the Arabidopsis circadian clock. *Plant Cell* 23 486–498. 10.1105/tpc.110.081661 21357491 PMC3077782

[B38] Wanke D. Hohenstatt M. L. Dynowski M. Bloss U. Hecker A. Elgass K. (2011). Alanine Zipper-Like Coiled-Coil Domains Are Necessary for Homotypic Dimerization of Plant GAGA-Factors in the Nucleus and Nucleolus. *PLoS One* 6:e16070. 10.1371/journal.pone.0016070 21347358 PMC3037368

[B39] Winter D. Vinegar B. Nahal H. Ammar R. Wilson G. V. Provart N. J. (2007). An “Electronic Fluorescent Pictograph” Browser for Exploring and Analyzing Large-Scale Biological Data Sets. *PLoS One* 2:e718. 10.1371/journal.pone.0000718 17684564 PMC1934936

[B40] Wu J. Mohamed D. Dowhanik S. Petrella R. Gregis V. Li J. (2020). Spatiotemporal Restriction of FUSCA3 Expression by Class I BPCs Promotes Ovule Development and Coordinates Embryo and Endosperm Growth. *Plant Cell* 32 1886–1904. 10.1105/tpc.19.00764 32265266 PMC7268797

[B41] Zielinski T. Moore A. M. Troup E. Halliday K. J. Millar A. J. (2014). Strengths and Limitations of Period Estimation Methods for Circadian Data. *PLoS One* 9:e96462. 10.1371/journal.pone.0096462 24809473 PMC4014635

[B42] Zuo J. Hare P. D. Chua N. H. (2006). Applications of chemical-inducible expression systems in functional genomics and biotechnology. *Methods Mol. Biol.* 323 329–342. 10.1385/1-59745-003-0:329 16739588

