# Supplementary material for: Family Members Additively Repress the Ectopic Expression of BASIC PENTACYSTEINE3 to Prevent Disorders in Arabidopsis Circadian Vegetative Development
Source: Front Plant Sci. 2022 May 26;13:919946. doi: 10.3389/fpls.2022.919946 (PMC9182635; doi:10.3389/fpls.2022.919946)
Supplement: Supplementary file 1 [file Presentation_1.pdf]

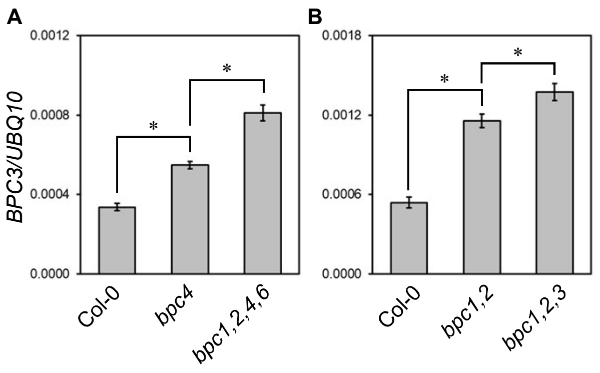

Supplementary Figure 1 | The second independent biological replicate of **Figure 2A (A)** and **2B (B)**.

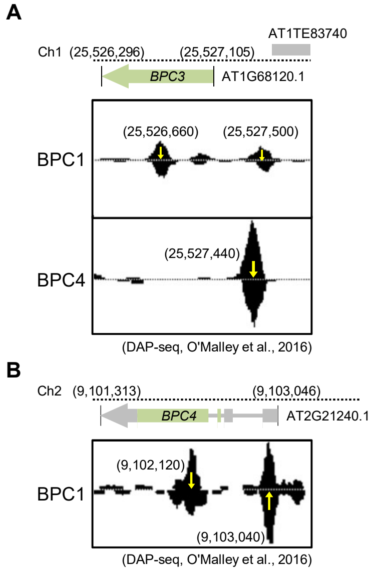

Supplementary Figure 2 | BPC members target *BPC3* and *BPC4* loci in the DAP-seq database. **(A,B)** A browser view of the public DAP-seq database shows histograms of DAP signals of BPC1 and BPC4 aligning at *BPC3* **(A)** and *BPC4* **(B)** loci (O'Malley et al., 2016). Numbers in parentheses indicated nucleotide positions at the chromosomes or positions of DAP-peaks indicated by yellow arrows. Gray and green boxes are respective exons and coding DNA sequences (CDS) of indicated genes.

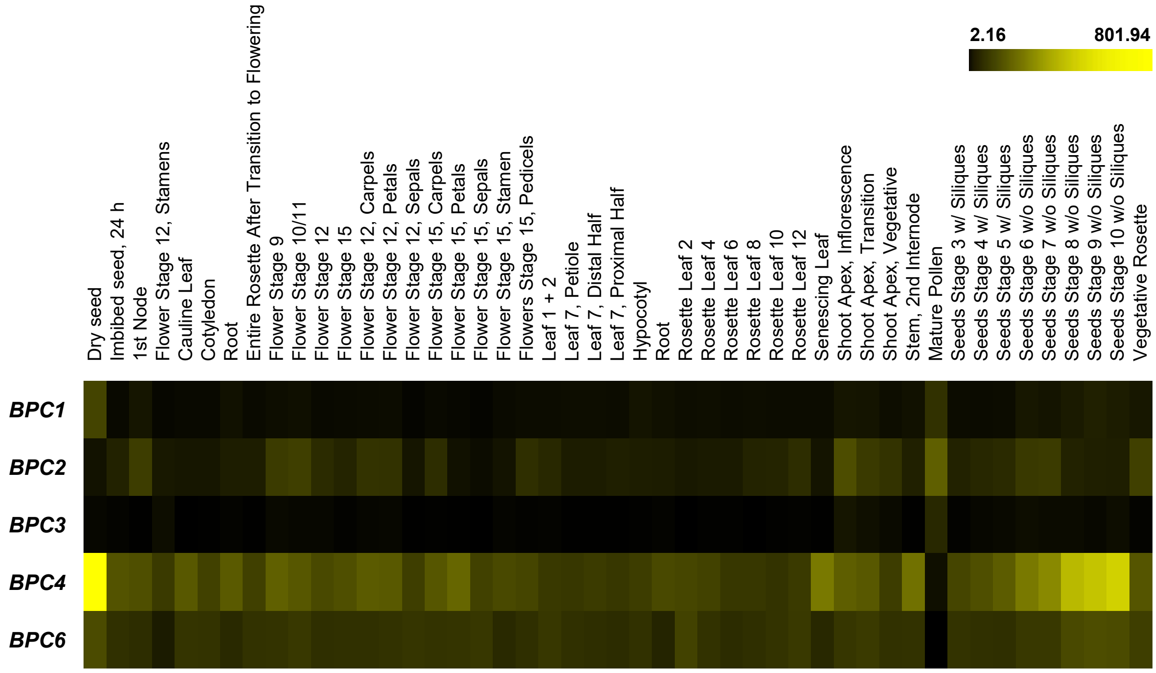

Supplementary Figure 3 | *BPC4* transcript level is higher than other *BPCs* across most developmental conditions. The meta-information from NASCArrays experiment 153 hosted by Arabidopsis microarray eFP browser (http://bar.utoronto.ca/efp/cgi-bin/efpWeb.cgi) were obtained as absolute values for *BPC1*, *BPC2*, *BPC3*, *BPC4*, and *BPC6* profiles across developmental series in wild type Columbia (Col-0).

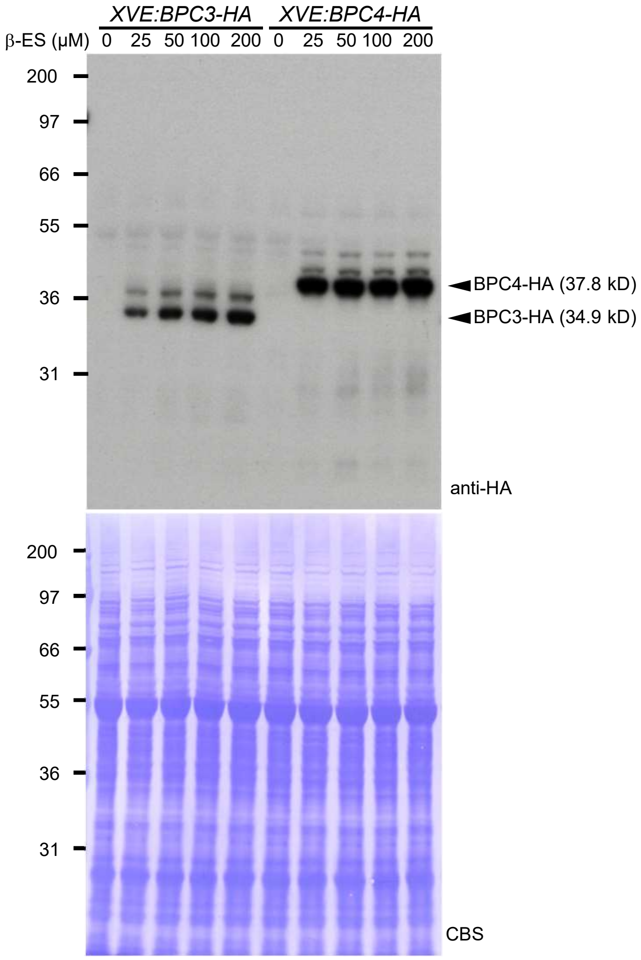

Supplementary Figure 4 | The induction of BPC3-HA and BPC4-HA in the *XVE:BPC3-HA* and *XVE:BPC4-HA* transgenic plants. Twelve-d-old plants of *XVE:BPC3-HA* and *XVE:BPC4-HA* transgenic lines were treated the indicated concentrations of 17-β-estradiol (β-ES) for one day. The protein levels of BPC3-HA and BPC4-HA in transgenic plants under the indicated concentrations of β-ES were determined by western blot analyses with anti-HA antibody. Specific proteins with putative sizes of BPC3-HA and BPC4-HA (arrow head) was induced along with inducer treatments. Coomassie blue staining (CBS) shows the protein amount on the blot.

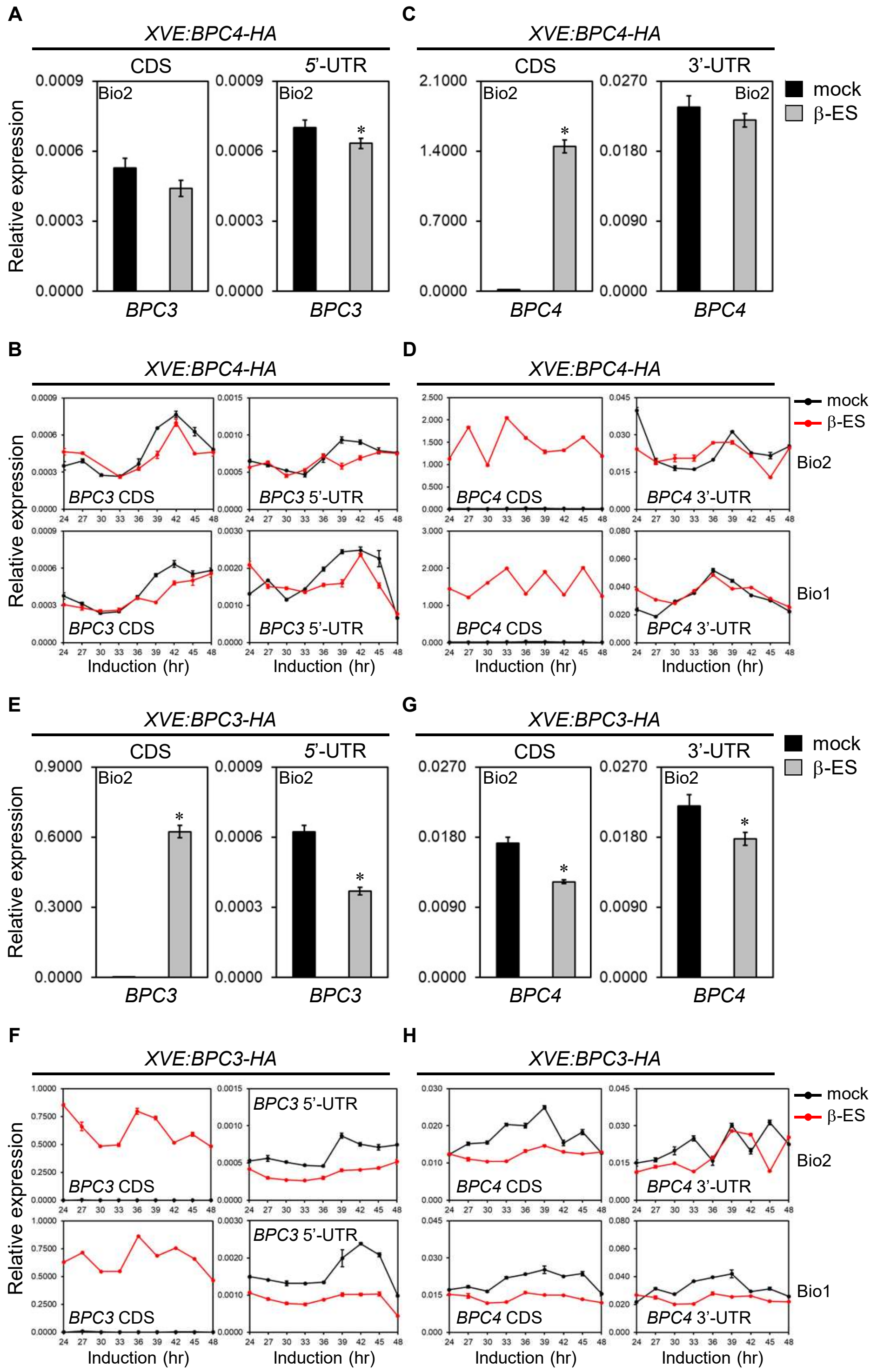

Supplementary Figure 5 | The independent biological replicate of data shown in **Figure 3**. The second independent biological replicate and corresponding individual time points used for average expression level calculations shown in **Figure 3A (A,B)**, **Figure 3B (C,D)**, **Figure 3C (E,F)**, **Figure 3D (G,H)**. The biological replicate shown in **Figure 3** is Bio1. Asterisks indicated transcript levels were significantly changed under b-estradiol treatments (Student's *t* test; $^{*}P < 0.05$).

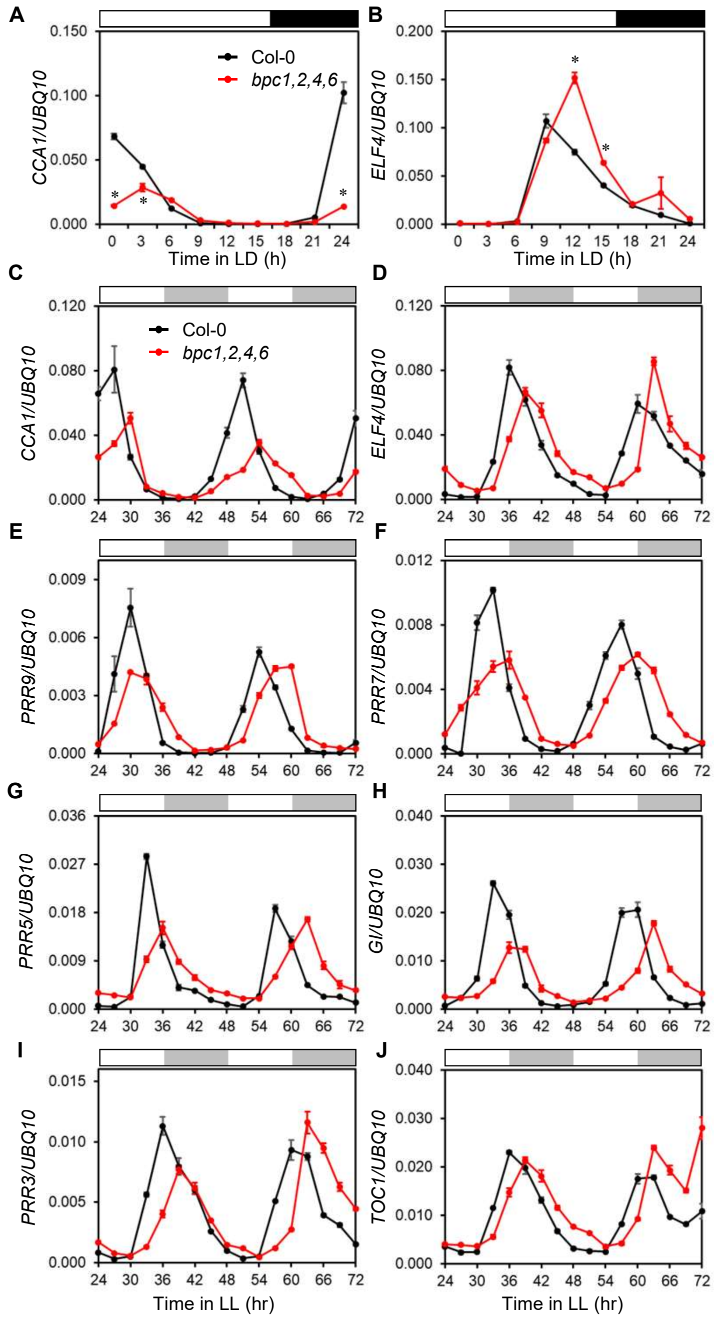

Supplementary Figure 6 | The independent biological replicate of data shown in **Figure 4**.

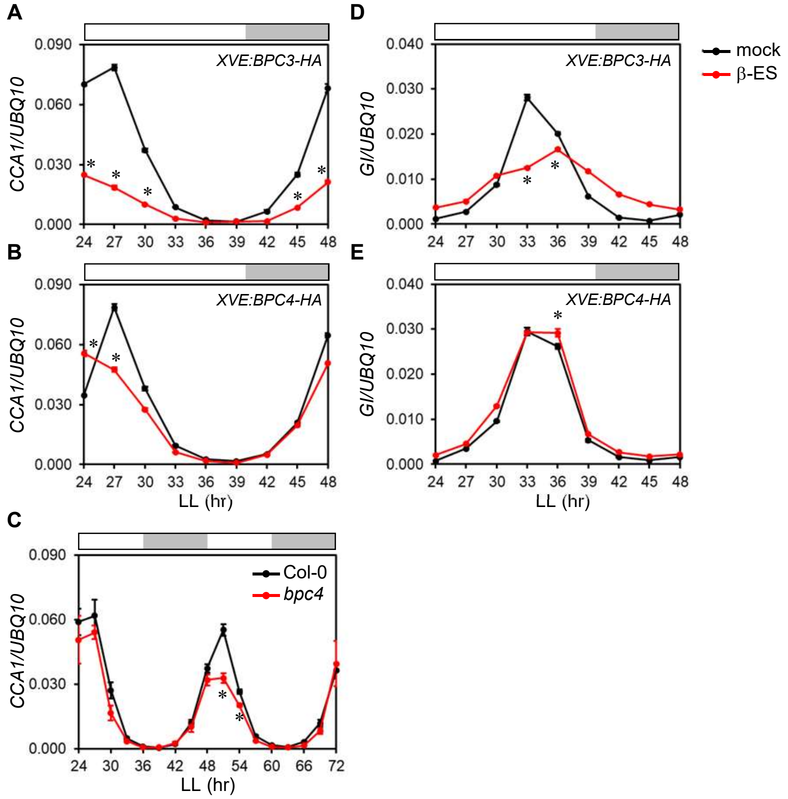

Supplementary Figure 7 | The independent biological replicate of data shown in **Figure 6**. **(A,B)** The second independent biological replicate of **Figure 6A** and **6B (A,B)**. **(C)** Eighteen-d-old plants of wild type (Col-0) and *bpc4* were transferred to the constant light (LL) and harvested at 3-h intervals from LL24h to LL72h for *CCA1* profiling by qRT-PCR analyses. Data are mean ± s.e. (n = 4 technical replicates of two independent biological replicates). The asterisk indicated the expression level was significantly decreased in *bpc4* (Student's *t* test; **P* < 0.005). **(D,E)** The second independent biological replicate of **Figure 6C (D)** and **6D (E)**.

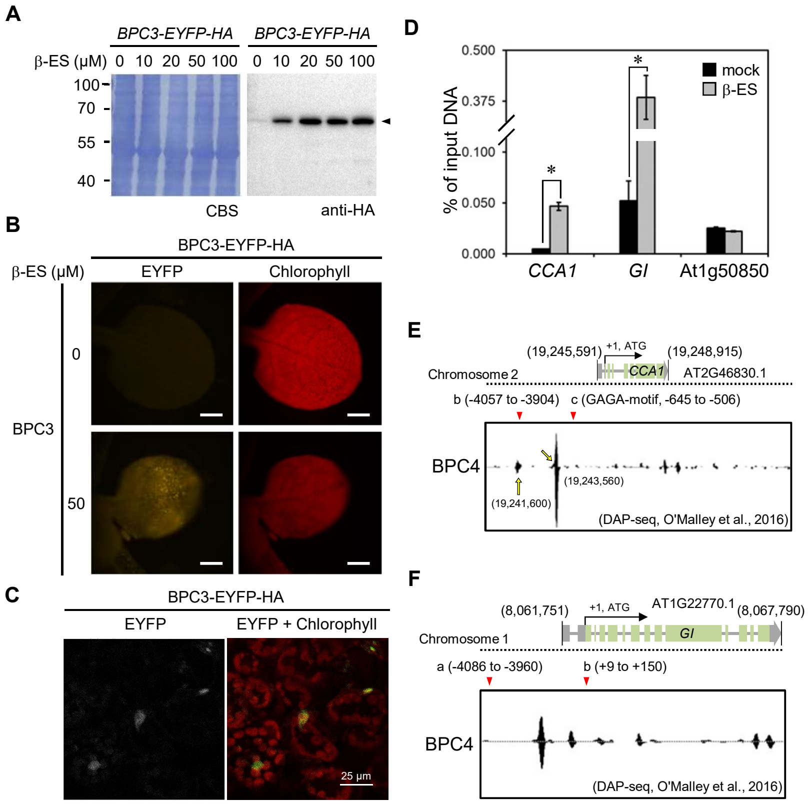

Supplementary Figure 8 | The induction of BPC3-EYFP-3HA for ChIP-qPCR assays. **(A)** The EYFP-HA-tagged BPC3 (BPC3-EYFP-HA) in transgenic seedlings under 24-h induction by 17-β-estradiol (β-ES) at indicated concentrations were analyzed by western blot analysis with anti-HA antibody. The arrow head indicated signals of BPC3-EYFP-HA with the predicted size 67.5 kD. The coomassie blue stained blot (CBS) was shown for protein loading. **(B)** The transgenic seedlings treated with 0 or 50 mM β-ES line was observed under the fluorescent microscopy to detect EYFP signal (Bar = 200 μm). The chlorophyll fluorescent signal was shown for cotyledon shape. **(C)** EYFP emission from the induced seedlings was recorded at 527 nm (left panel) and merged with 650-nm chlorophyll emission by using a laser spectroscope (Photonic Workshop, Center for Condensed Matter Sciences, National Taiwan University). **(D)** An independent biological replicate for ChIP-qPCR assays with amplicon 'c' of *CCA1*, 'b' of *GI*, and the transposon control (Atg50850) in **Figure 6E** and **6F**. **(E,F)** Browser view of public DAP-seq database shows histograms of DAP signals of BPC4 aligning at *CCA1* **(E)** and *GI* **(F)** loci (O'Malley et al., 2016).

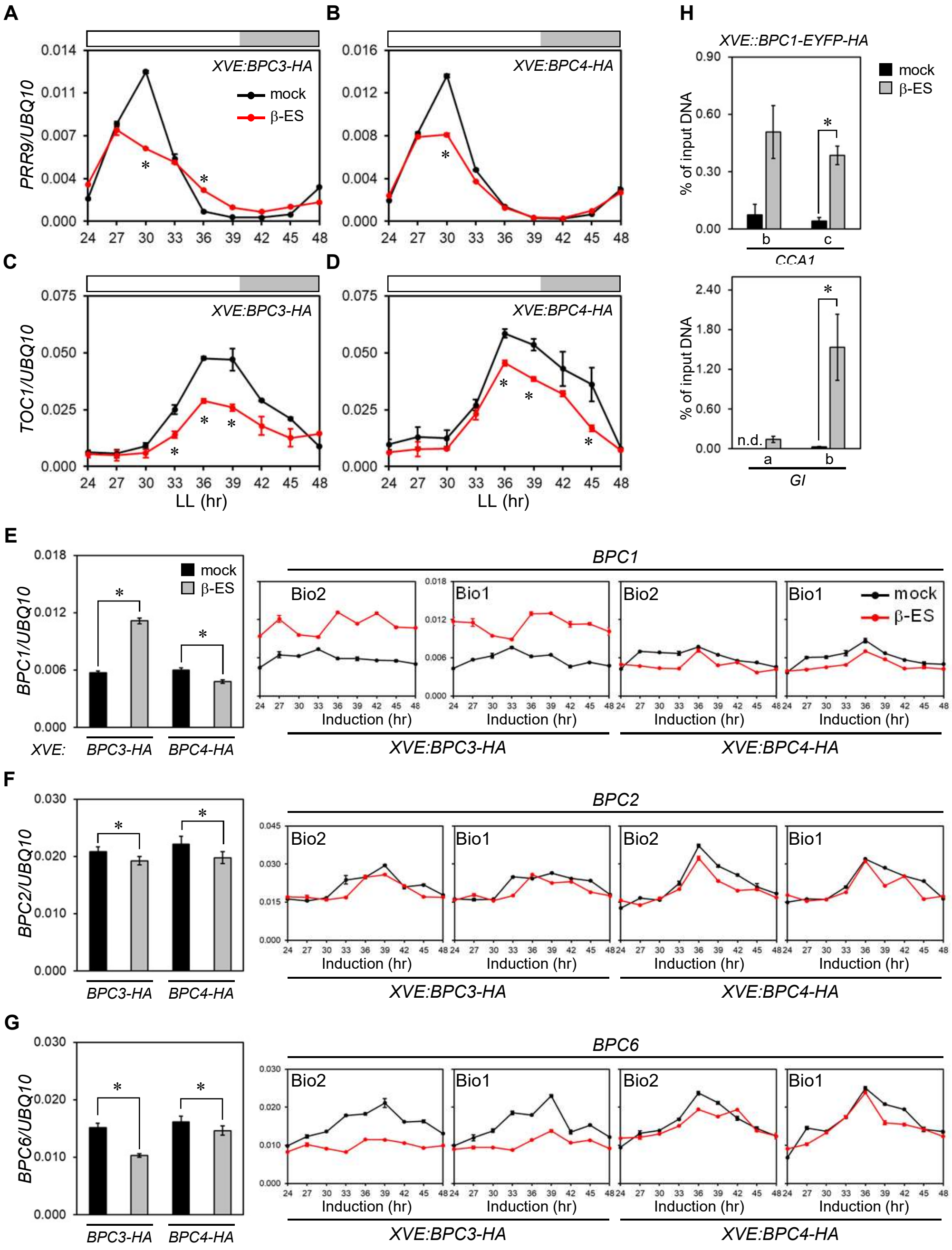

Supplementary Figure 9 | The independent biological replicate of data shown in **Figure 7**. **(A–D)** The second independent biological replicate of **Figure 7A–D**. **(E–G)** The second independent biological replicate and corresponding individual time points used for average expression level calculations in **Figure 7E–G**. The biological replicate shown in **Figure 7** is Bio1. **(H)** ChIP-qPCR assays conducted as described in **Figure 6E** and **6F** by using transgenic *XVE:BPC1-EYFP-HA* lines.

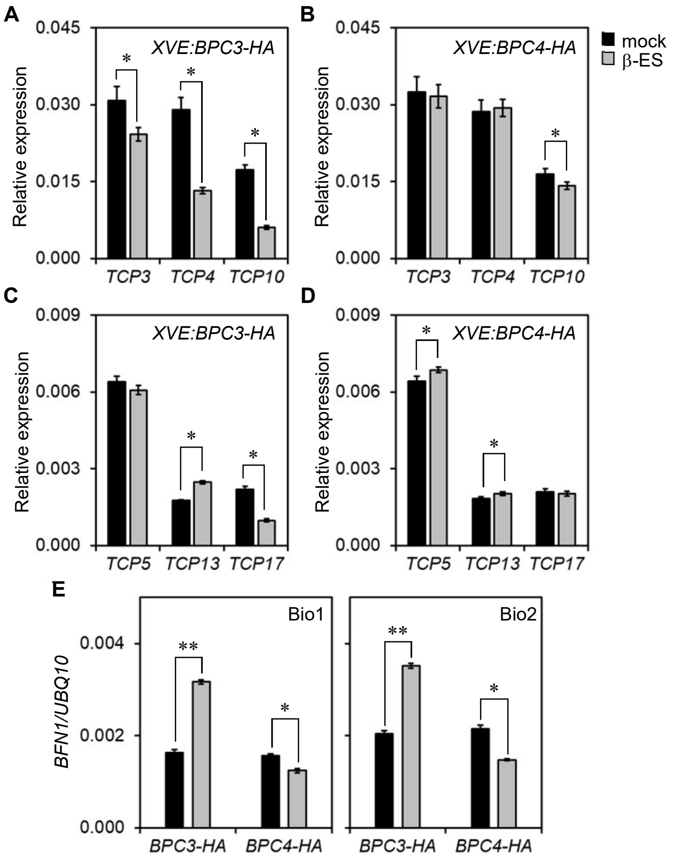

Supplementary Figure 10 | Genes involved in edge formation and leaf senescence are regulated by BPC3 and BPC4. **(A–D)** The independent biological replicate of **Figure 8C (A)**, **8D (B)**, **8E (C)**, and **8F (D)**. **(E)** The expressions of *BFN1* relative to that of *UBQ10* were analyzed in *XVE:BPC3-HA* or *XVE:BPC4-HA* transgenic plants under 0 (mock) or 50 mM 17-β-estradiol (β-ES) inductions. Data are mean ± s.e. (n = 27, data collected as described in **Figure 3**). Asterisks indicated expressions significantly changed by the β-ES (Student's *t* test; **P* < 0.05, ***P* < 0.01). The transcript levels of individual time points were shown in **Supplementary Figure 11**.

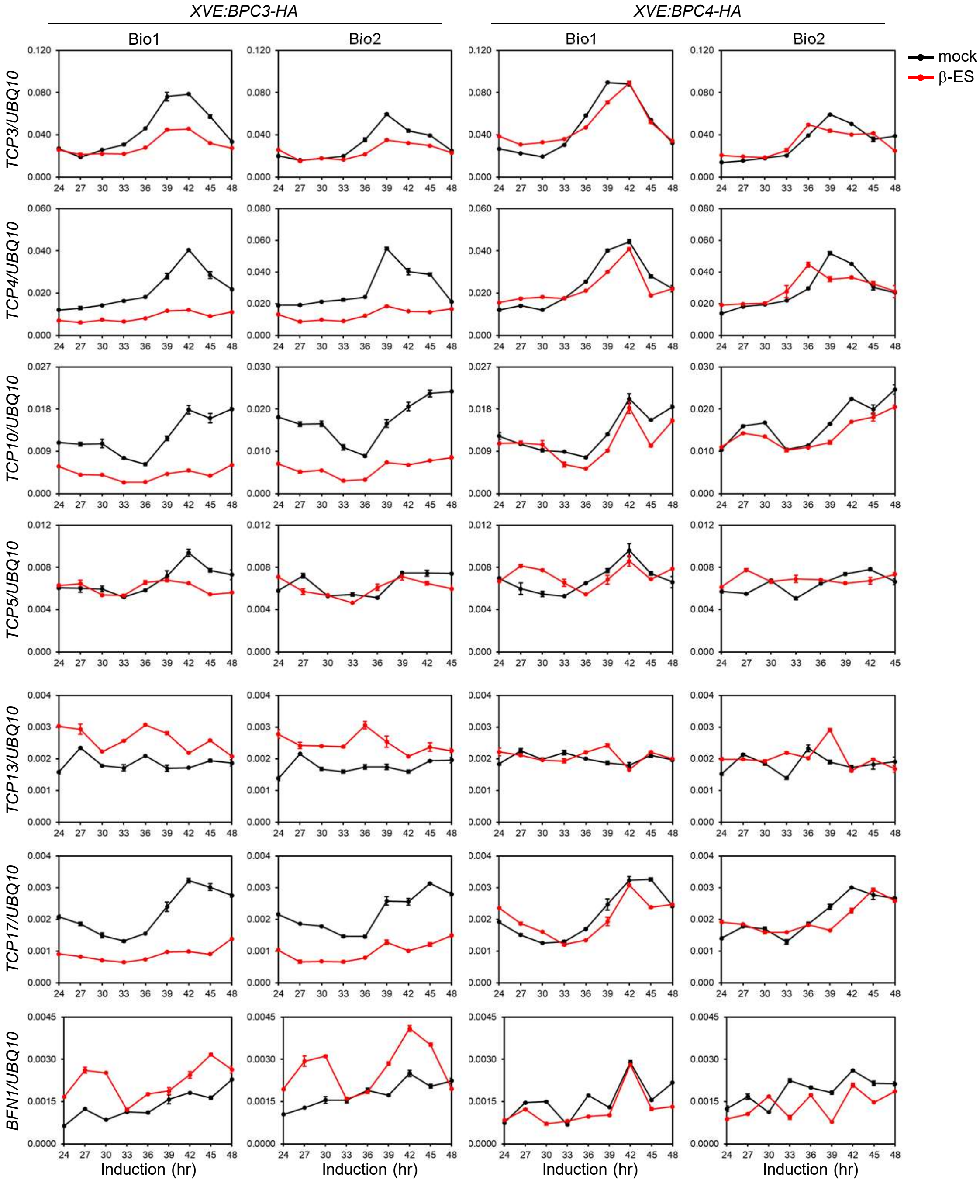

Supplementary Figure 11 | Individual time points for BPC3-HA and BPC4-HA induction effects on gene expressions. The qRT-PCR results of individual time points used in average expression profiles of *TCP3*, *TCP4*, *TCP10*, *TCP5*, *TCP13*, *TCP17*, and *BFN1* in **Figure 8** and **Supplementary Figure 10**.
